# Biosynthesis of the antifungal haterumalide, oocydin A, in *Serratia*, and its regulation by quorum sensing, RpoS and Hfq

**DOI:** 10.1111/1462-2920.12839

**Published:** 2015-04-08

**Authors:** Miguel A Matilla, Finian J Leeper, George P C Salmond

**Affiliations:** 1 Department of Biochemistry, University of CambridgeTennis Court Road, Cambridge, CB2 1QW, UK; 2 Department of Chemistry, University of CambridgeLensfield Road, Cambridge, CB2 1EW, UK

## Abstract

Polyketides represent an important class of bioactive natural products with a broad range of biological activities. We identified recently a large *trans*-acyltransferase (AT) polyketide synthase gene cluster responsible for the biosynthesis of the antifungal, anti-oomycete and antitumor haterumalide, oocydin A (*ooc*). Using genome sequencing and comparative genomics, we show that the *ooc* gene cluster is widespread within biocontrol and phytopathogenic strains of the enterobacteria, *Serratia* and *Dickeya*. The analysis of in frame deletion mutants confirmed the role of a hydroxymethylglutaryl-coenzyme A synthase cassette, three flavin-dependent tailoring enzymes, a free-standing acyl carrier protein and two hypothetical proteins in oocydin A biosynthesis. The requirement of the three *trans*-acting AT domains for the biosynthesis of the macrolide was also demonstrated. Expression of the *ooc* gene cluster was shown to be positively regulated by an *N*-acyl-L-homoserine lactone-based quorum sensing system, but operating in a strain-dependent manner. At a post-transcriptional level, the RNA chaperone, Hfq, plays a key role in oocydin A biosynthesis. The Hfq-dependent regulation is partially mediated by the stationary phase sigma factor, RpoS, which was also shown to positively regulate the synthesis of the macrolide. Our results reveal differential regulation of the divergently transcribed *ooc* transcriptional units, highlighting the complexity of oocydin A production.

## Introduction

Streptomycetes and related actinomycetes continue to be the main source of novel secondary metabolites, providing more than half of the clinically used antibiotics and anticancer agents (Liu *et al*., 2013). However, with increasing numbers of genomes sequenced and the improvement of bioinformatic, genetic and analytical tools, it has become more apparent that there is widespread distribution of polyketide synthase (PKS) and non-ribosomal peptide synthetase (NRPS) biosynthetic gene clusters within the bacterial kingdom (Hertweck, 2009; Piel, 2010; Helfrich *et al*., 2014; Wang *et al*., 2014).

With an estimated worldwide annual sales of $20 billion (Koryakina *et al*., 2013), bioactive polyketides show a broad range of important clinical and agricultural applications and represent a key class of natural products (Hertweck, 2009; Piel, 2010; Till and Race, 2014). Type I PKSs are usually large multidomain proteins in which the domains are organized in PKS modules, each of which is responsible for a round of polyketide chain elongation and processing. The classical PKS extension module consists of an acyl carrier protein (ACP) where the chain is assembled and elongated; an acyltransferase (AT) responsible for selecting and transferring short coenzyme A (CoA)-activated extender units to the ACP domain; and a ketosynthase (KS) that catalyses the chain elongation reaction. This minimal KS-AT-ACP module can be supplemented with additional domains such as ketoreductases, enoyl reductases (ER), methyltransferases (MT) and dehydratases (DH) that are responsible, along with other post-assembly tailoring reactions, for much of the structural diversity of polyketides (Hertweck, 2009; Piel, 2010).

The halogenated macrolide, oocydin A (Fig. S1), was identified in the late 1990s from a plant epiphytic bacterial strain of *Serratia marcescens* (Strobel *et al*., 1999) and from the sponge, *Ircinia* sp. (Takada *et al*., 1999). Oocydin is a member of the haterumalide class of molecules, and it has potent bioactivity against plant pathogenic oomycetes, but it also shows anticancer properties. The same macrolide was subsequently isolated from other bacterial strains and was shown to have antifungal (Thaning *et al*., 2001) and anti-hyperlipidemic (Sato *et al*., 2005) activities. Recently, the *trans*-AT PKS gene cluster responsible for the biosynthesis of oocydin A was identified and a model for its biosynthesis proposed (Matilla *et al*., 2012). *Trans*-AT PKSs represent around 3.8% of all sequenced gene clusters (Wang *et al*., 2014) and they are characterized by a lack of AT domains in the PKS modules, with the ACP domains being instead loaded by free-standing AT enzymes (Piel, 2010). The majority of the *trans*-AT systems possess numerous functional peculiarities which often result in unusual chemistry (Piel, 2010). Some of these peculiarities, including uncommon domain orders and splitting of modules between PKS proteins, were found in the oocydin A (*ooc*) gene cluster (Matilla *et al*., 2012) and these traits make the investigation of the corresponding biochemistry a stimulating challenge.

The biological production of secondary metabolites can be energetically costly and so it is usually highly regulated (Coulthurst *et al*., 2005; Williamson *et al*., 2006; Liu *et al*., 2013). One environmental input to regulation of some natural products is quorum sensing (QS). Through QS, bacteria can sense their population density and regulate target gene expression accordingly, via the production and detection of diffusible autoinducer signalling molecules. In Gram-negative bacteria, the most common QS systems are those that employ *N*-acyl-L-homoserine lactones (AHLs) as the autoinducer molecules. Generally, AHLs are synthesized by members of the LuxI family of AHLs synthases. When a critical AHL threshold concentration is reached, the interaction between the autoinducer and a LuxR-type transcriptional regulator results in the alteration of the expression of QS-dependent genes (Van Houdt *et al*., 2007a; Atkinson and Williams, 2009).

Biosynthesis of secondary metabolites can be also controlled at the post-transcriptional level, for example through the RNA binding proteins CsrA (Romeo *et al*., 2013) and Hfq (Vogel and Luisi, 2011). The chaperone Hfq acts by facilitating base pairing of regulatory small RNAs (sRNAs) and cognate target mRNAs. Thus, Hfq can modulate translation rate, affect the lifetime of the targeted transcripts or provoke changes in RNA structure (Vogel and Luisi, 2011). Hfq is present in at least half of the currently sequenced bacterial genomes (Vogel and Luisi, 2011) and regulates the expression of 4–15% of the total bacterial transcriptome (Ding *et al*., 2004; Guisbert *et al*., 2007; Sittka *et al*., 2008; Wilf *et al*., 2013). Numerous studies have also demonstrated the *in vivo* interaction of Hfq with sRNAs and mRNAs (Wagner, 2013).

In this study, we tested our recent model for the biosynthesis of oocydin A (Matilla *et al*., 2012) through a comprehensive in frame deletion mutagenesis of specific *ooc* non-PKS encoding genes in the biocontrol rhizosphere bacterium, *Serratia plymuthica* A153. The regulation of oocydin A production was also investigated in several other enterobacterial strains and the results show that the expression of the *ooc* gene cluster is controlled at transcriptional and post-transcriptional levels.

## Results

### The oocydin A gene cluster is widely dispersed within the plant pathogenic *Dickeya* genus, and other plant-associated enterobacteria

**Fig. 1:**
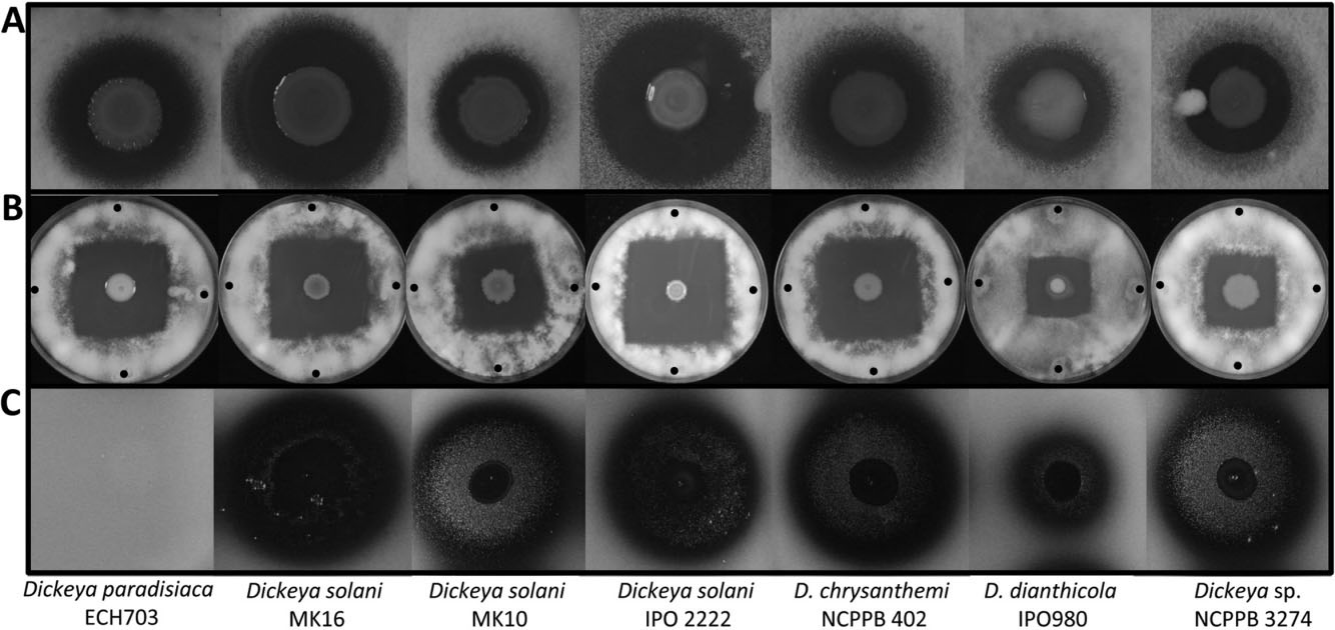
Bioactivity and *N*-acyl homoserine lactone (AHL) signalling molecule production in oocydin A-producing *Dickeya* strains. Bioactivities against *Verticillium dahliae* (A) and *Pythium ultimum* (B) are shown. Black dots indicate *P. ultimum* inoculation points. (C) Violacein production by the biosensor strain *Chromobacterium violaceum* CV026 in response to AHLs produced by the *Dickeya* strains*.* In all cases, 5 μl of an overnight culture of the selected strains were spotted on the surface of the bioassay plates. The bioassays were repeated at least three times, and representative results are shown. CV026, *P. ultimum* and *V. dahliae* pictures were taken after 48, 72 and 96 h of incubation at 25°C respectively.

Previously, we showed that the *ooc* gene cluster is present in three plant-associated *Serratia* strains and a strain of *Dickeya* (Matilla *et al*., 2012). However, the genomes of multiple new *Dickeya* strains were sequenced recently (Garlant *et al*., 2013; Pritchard *et al*., 2013a,b[Bibr b53],[Bibr b54]; Khayi *et al*., 2014) and our genomic analyses revealed that the *ooc* gene cluster is present in around half of them, including *Dickeya solani* strains MK10, MK16, IPO 2222, 3337, D-s0432-1 and GBBC 2040; *Dickeya dianthicola* strains NCPPB 453, NCPPB 3534, GBBC 2039 and IPO 980; *Dickeya chrysanthemi* NCPPB 402; *Dickeya paradisiaca* NCPPB 2511; *Dickeya* sp. CSL RW240 and *Dickeya* sp. NCPPB 3274 (Fig. S2). We had access to these *Dickeya* strains and, as predicted, they showed strong antifungal and anti-oomycete activities (Fig. 1A and B). Comparative analyses showed that the *ooc* gene clusters encoded by the *Dickeya* strains are between 78.2% and 100% identical at the DNA level (Fig. S2 and Table S1).

Subsequently, we re-screened the antifungal and anti-oomycete activities of several enterobacterial strains isolated from the rhizosphere of economically important crops (Berg *et al*., 2002). Strains belonging to *Serratia*, *Pantoea* and *Xenorhabdus* genera showed the same bioactivities as other oocydin A producers and polymerase chain reaction (PCR) analyses confirmed these were new candidate oocydin A-producing strains (Fig. S3). Genome sequencing of one strain, *S. plymuthica* 4Rx5 (M. A. Matilla and G. P. C. Salmond, unpublished), confirmed that the complete *ooc* gene cluster is present in this rhizobacterium (Fig. S4).

### OocA and OocB are not required for the synthesis and secretion of oocydin A

**Fig. 2:**
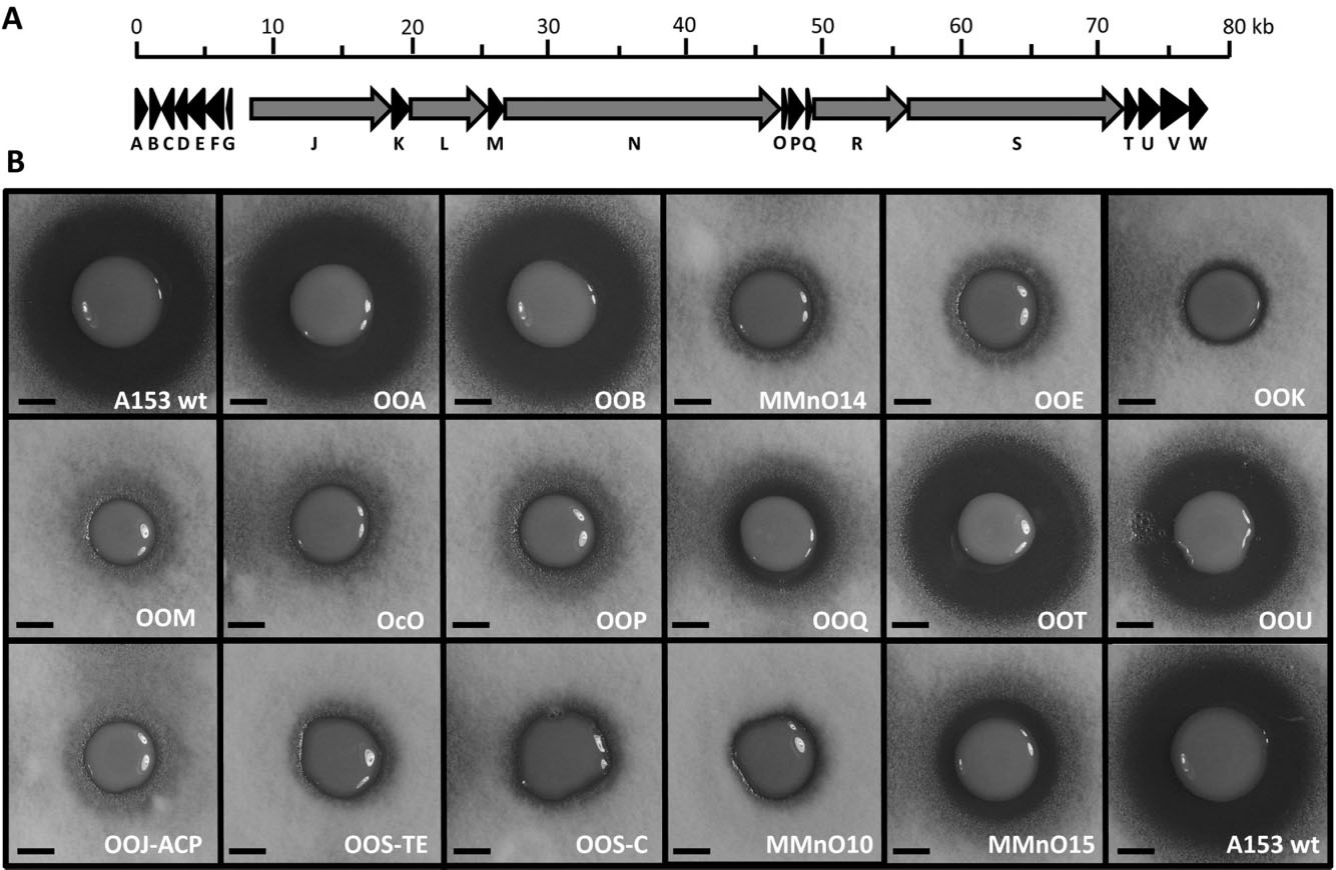
Antifungal activities of *Serratia plymuthica* A153 and derivative strains with mutations in the *ooc* biosynthetic gene cluster.A. Gene organization of the *ooc* gene cluster in *Serratia* strains. Modular PKS encoding genes are shown in grey.B. Bioactivities of *S. Plymuthica* A153 strains against *Verticillium dahliae* are shown. The bioassays were repeated at least three times, and representative results are shown. MmnO10 and MmnO15 produce none and 3–5% of the oocydin A wild type levels respectively (Matilla *et al*., 2012). Pictures were taken after 96 h of incubation at 25°C. Bioactivities against the oomycete *Pythium ultimum* are shown in Fig. S5. In frame deletions were complemented by expressing the genes *in trans* using pQE-80L-based vectors (Fig. S6). Bars, 5 mm.

The *ooc* gene cluster is organized in three transcriptional units, the first of which encodes the α/β-Hydrolase, OocA, and the putative efflux protein, OocB (Fig. 2A). The *oocA* gene is conserved in the sequenced oocydin A-producing *S. plymuthica* strains A153, 4Rx5 and 4Rx13, and *S. marcescens* MSU97. However, *oocA* is absent from all the *ooc* gene clusters in the *Dickeya* strains – including Ech703, a strain we showed previously to produce oocydin A (Matilla *et al*., 2012). To determine whether OocA and OocB affect the biosynthesis and secretion of oocydin A, we constructed in frame deletion mutants defective in *oocA* and *oocB* in *S. plymuthica* A153. The production of oocydin A in the derivative strains (OOA and OOB) was assessed by bioassays using the oomycete, *Pythium ultimum*, and the fungal phytopathogen, *Verticillium dahliae.* The OOA strain exhibited the same antifungal (Fig. 2B) and anti-oomycete (Fig. S5) activities as the wild type strain. Surprisingly, given its conservation within the *Serratia* and *Dickeya* genera, the in frame deletion of *oocB* did not alter the bioactive properties of A153 either (Figs 2B and S5). This result may indicate that *oocB* is not involved in oocydin A secretion or that another secretion system encoded in the genome of A153 could also perform this function.

### Requirement of non-PKS proteins for the biosynthesis of oocydin A

To gain insights into the biosynthesis of oocydin A and the role of the Ooc proteins, in frame deletion mutants defective in the putative tailoring enzymes and non-multidomain PKS encoding genes were constructed, thereby avoiding polar effects on the transcription of the downstream genes. The generated mutants were tested in dual culture assays (Figs 2B and S5) and functionally complemented *in trans* (Fig. S6).

The second transcriptional unit of the *ooc* gene cluster consists of five genes, *oocG-C*, encoding a 3-hydroxy-3-methylglutaryl-CoA synthase (HCS) cassette proposed to be responsible for introducing a methyl group at C-17 of oocydin A (Matilla *et al*., 2012). In support of this hypothesis, a transposon mutant defective in the last gene of the HCS cassette of the *ooc* gene cluster encoding the enoyl-CoA hydratase (OocC), and an in frame deletion mutant defective in the hydroxymethylglutaryl-CoA synthase-encoding gene (*oocE*), displayed a complete loss of oocydin A production (Figs 2B and S5).

The PKS core machinery for the biosynthesis of oocydin A (the multidomain PKS enzymes OocJ, OocL, OocN, OocR and OocS) is encoded by a third transcriptional unit in the *ooc* gene cluster. This transcriptional unit also encodes three flavin-dependent enzymes (OocK, OocM and OocU), three hypothetical proteins (OocP, OocQ and OocT) and the free-standing ACP, OocO. To determine whether these proteins were required for oocydin A production, in frame deletion mutants were first constructed by allelic exchange. The corresponding mutant strains OOK, OOM, OcO and OOP no longer exhibited antimicrobial activity against *V. dahliae* (Fig. 2B) or *P. ultimum* (Fig. S5). The flavin-dependent monooxygenases OocK and OocM are predicted to catalyse the hydroxylation and the halogenation required to produce oocydin A. However, no dechloro analogues of oocydin A were detected in the extracts of OOK or OOM (not shown), perhaps suggesting that the halogenation may occur on an intermediate covalently attached to the PKS rather than at a post-PKS biosynthesis step. The loss of oocydin A production in Δ*oocO* indicates the essential role of this ACP in the biosynthesis of the halogenated macrolide (Fig. 2B). The presence of free-standing ACPs is not uncommon within PKS gene clusters (Piel, 2010; Cantu *et al*., 2012) including *trans*-AT PKS clusters such as those for mupirocin (El-Sayed *et al*., 2003; Gurney and Thomas, 2011) and difficidin (Chen *et al*., 2006). Given that the catalytic Cys-His-His triad is absent in the last KS domain of OocN, we suggest that this KS may be responsible for transferring the acyl intermediate to the ACP OocO without extending it, as an intermediate step involved in transferring the biosynthetic intermediate to the first ACP domain of the PKS, OocR. Alternatively, the full-length polyketide chain could be offloaded from the PKS OocS and transferred to the discrete ACP OocO for further processing by tailoring enzymes, as previously proposed for the biosynthesis of salinomycin (Yurkovich *et al*., 2012) and monensin (Hüttel *et al*., 2014).

The protein OocU shows high similarity to the PKS biosynthetic enzymes, DifA (Chen *et al*., 2006), PksE (Bumpus *et al*., 2008) and BatK (Mattheus *et al*., 2010). PksE was reported to possess *trans*-ER activity *in vitro* (Bumpus *et al*., 2008) and in frame deletion of *batK* eliminated biosynthesis of the polyketide kal/bat (Mattheus *et al*., 2010). Our oocydin A biosynthetic model suggested that an ER is required for the synthesis of the final polyketide (Matilla *et al*., 2012). However, an in frame deletion of *oocU* only caused a reduction in the bioactive properties of A153 (Figs 2B and S5) suggesting that another ER encoded in the *ooc* gene cluster or elsewhere in the A153 genome could be catalysing this reaction. Interestingly, the in frame deletion of *oocP* and *oocQ* resulted in mutants showing no, or very low, bioactivity, respectively, revealing the importance of these proteins of unknown function in the synthesis of oocydin A (Figs 2B and S5). In contrast, an in frame deletion of *oocT* showed similar bioactivity to that of the wild type strain, A153 (Figs 2B and S5). Bioinformatic analyses did not shed light on the role of OocP and OocQ, but a predicted cupin-like domain was found in OocP. The low sequence similarity exhibited between cupin proteins makes it difficult to predict their functional roles, although cupin-like proteins such as BacB (Rajavel *et al*., 2009) and MomA (Zeng *et al*., 2012) have been found associated with NRPS and PKS biosynthetic gene clusters respectively.

### Point mutations confirm the role of ACP_L_, TE and NRPS-C domains in the biosynthesis of oocydin A

FkbH domains have been shown to be responsible of loading unusual biosynthetic extender units onto ACP domains (Sun *et al*., 2008) and an FkbH-like domain is present in the first module of the PKS OocJ, DH-MT-FkbH-ACP. However, the starter unit for the synthesis of oocydin A is predicted to be a malonyl unit, and the absence of the characteristic conserved motifs in the DH and MT domains (Matilla *et al*., 2012) suggests that this unconventional module of OocJ is not functional. On the other hand, the ACP domain of this module, ACP_L_, possesses the highly conserved G*X*DS signature motif (Aparicio *et al*., 1996). To test whether the ACP_L_ is required for the initiation of the polyketide chain growth, we constructed a site-directed mutant by changing the essential aspartic acid and serine residues (GVDS) to glycine and alanine (GV**GA**). The resulting mutant OOJ-ACP showed a complete loss of antifungal activity (Fig. 2B), indicating the important role of ACP_L_ for the synthesis of the polyketide.

Generally, after polyketide elongation, a C-terminal thioesterase (TE) domain is responsible for the macrolactonization that releases the polyketide from the PKS (Du and Lou, 2010). Interestingly, the last PKS of the *ooc* gene cluster, OocS, contains a TE and a NRPS condensation (NRPS-C) domain. These two domains are candidates for the final lactonization but also for the O-acetylation required (Matilla *et al*., 2012). In order to gain insights into the role of these domains in the biosynthesis of oocydin A, we first constructed the mutant strain OOS-TE in which we changed the active site serine residue of the TE motif, G*X*S*X*G (Konz and Marahiel, 1999), to alanine (GY**A**MG). A second strain, OOS-C, was generated by replacing the aspartic acid residue of the conserved NRPS-C motif HH*XXX*DG (Marahiel, 1997), essential for stabilizing the active site (Samel *et al*., 2007), to alanine (HHFHA**A**G). Neither mutant produced oocydin A, indicating the need for both domains in the generation of the final macrolide (Fig. 2B).

### Role of the *trans*-AT domains in the biosynthesis of oocydin A

**Fig. 3:**
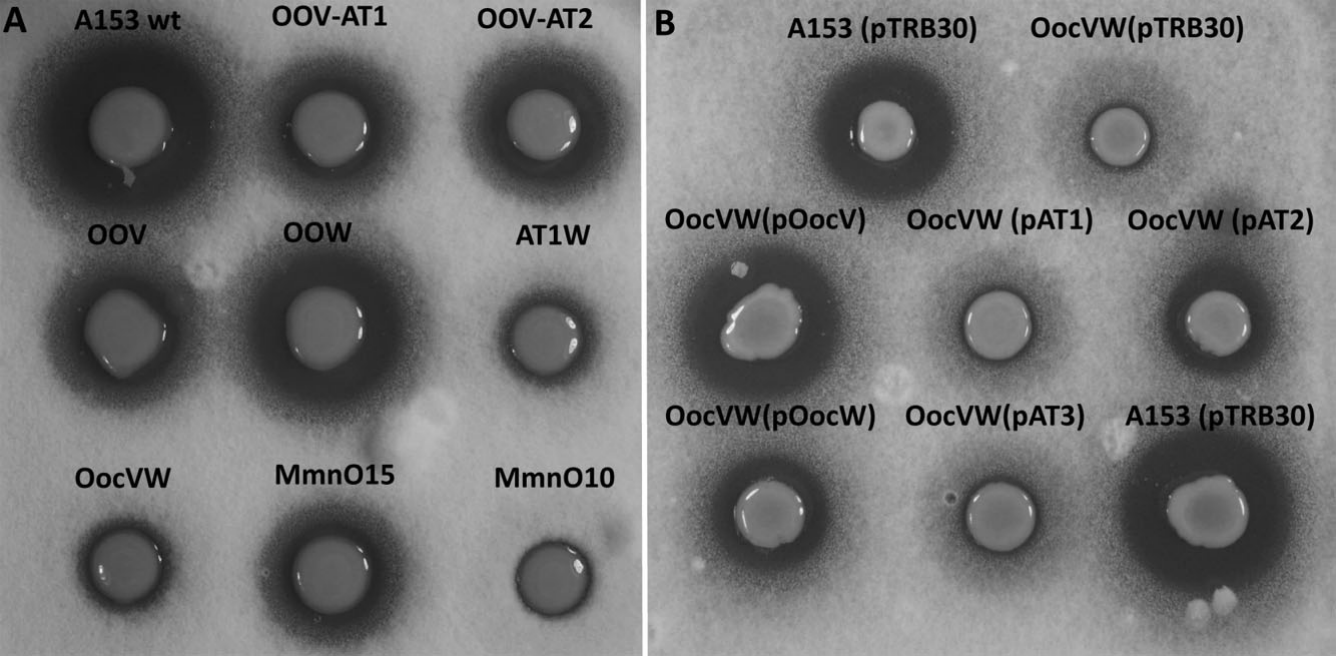
Role of the three *trans*-acyltransferase domains present in the *ooc* gene cluster for the biosynthesis of oocydin A.A. Bioactivities against *Verticillium dahliae* of *Serratia plymuthica* A153 mutants in the acyltransferase (AT) encoding genes, *oocV* and *oocW*. MmnO10 and MmnO15 produce none and 3–5% of the oocydin A wild type levels respectively (Matilla *et al*., 2012).B. Complementation of the strain OocVW by the *in trans* expression of OocV, OocW or alternatively OocV or OocW containing point mutations in the catalytic serine of their AT domains. Induction of the expression of the wild type and mutant proteins was done by addition of 1 mM of IPTG. The bioassays were repeated at least three times, and representative results are shown. Pictures were taken after 96 h of incubation at 25°C.

The *ooc* gene cluster encodes two discrete AT proteins, OocV and OocW, containing two (OocV-AT1 and OocV-AT2) and one (OocW-AT3) *trans*-AT domains, respectively, all with the catalytic Ser-His dyad (Matilla *et al*., 2012). To investigate the role of these domains in the biosynthesis of oocydin A, we performed mutagenesis and complementation analyses, and the strains were phenotypically characterized by their antifungal activities. An in frame deletion of *oocV* resulted in a dramatic diminution in bioactivity, comparable to the bioactivity levels of the mutant MMnO15 (Fig. 3A), which showed only 3–5% of the wild type levels (Matilla *et al*., 2012). Importantly, the deletion of OocV-AT1 resulted in a mutant with the same bioactivity as the mutant strain OOV (Fig. 3A). However, the deletion of the domain OocV-AT2 reduced the antifungal properties of the strain by around 50% in comparison with that of the wild type A153 (Fig. 3A). Perhaps unexpectedly, mutation of *oocW* caused only a slight reduction in bioactivity (Fig. 3A). However, the production of oocydin A was totally abolished in strain AT1W, which retains only an intact OocV-AT2 domain (Fig. 3A).

To clarify the role of the three AT domains in the biosynthesis of oocydin A, we constructed a non-bioactive strain, OocVW, in which both genes *oocV* and *oocW* were deleted. This strain was used for *in vivo* complementation assays in order to investigate whether single AT domains could restore oocydin A production. Wild type oocydin A levels were fully or partially restored by the expression of *oocV* and *oocW in trans* respectively (Fig. 3B). However, complementation was abolished when the catalytic serines of the domains OocV-AT1 or OocW-AT3 were mutated to alanine (Fig. 3B). Intriguingly, mutation of the catalytic serine of OocV-AT2 led to partial complementation of the defect in oocydin A production – to the same level as when *oocW* was expressed *in trans* in OocVW (Fig. 3B). These results indicate that OocV-AT1 is the main *trans*-AT domain involved in the biosynthesis of oocydin A but OocW-AT3 can substitute for it with lower efficiency if OocV-AT1 is missing or inactive. OocV-AT2 on its own cannot support oocydin A biosynthesis but may assist in some of the multiple acyl transfer steps.

### Transcription of the *ooc* gene cluster is growth phase dependent

Our mutational analyses showed that the genes essential for the biosynthesis of oocydin A are organized in two transcriptional units (Fig. 2). To investigate the transcription of the *ooc* gene cluster, we constructed two transcriptional fusions, P*_oocG_*::*lacZ* and P*_oocJ_*::*lacZ* in plasmids pMAMV165 and pMAMV166, respectively, and β-galactosidase activities expressed from these fusions in a Lac^–^ derivative of *S. plymuthica* A153 were assessed throughout growth.

**Fig. 4:**
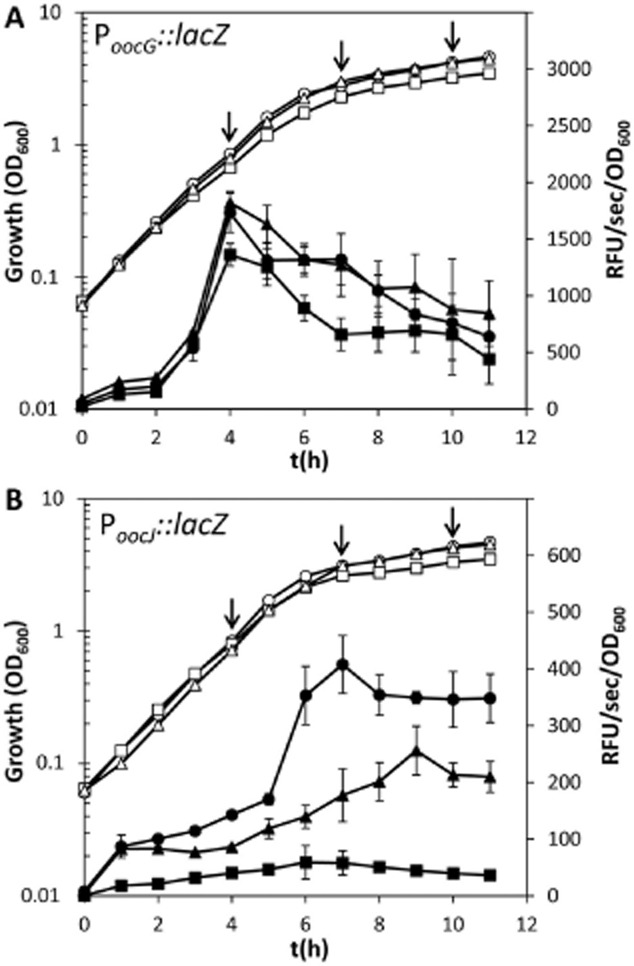
RpoS and Hfq regulate the expression of the oocydin A biosynthetic gene cluster. Transcription of the *oocG* (P*_oocG_::lacZ*; pMAMV165) (A) and *oocj* (P*_oocj_::lacZ*; pMAMV166) (B) promoter fusions throughout growth in *Serratia plymuthica* A153 strains. β-Galactosidase activity (filled symbols) and growth curves (open symbols) were determined in LacZ (circles), and its *hfq* (AHfqL; squares) and *rpoS* (RpoSL; triangles) derivative strains in LB medium at 25°C. Data are the mean and standard deviation of three biological replicates. Arrows, time points when samples for qPCR were taken.

Transcription from the *oocG* and *oocJ* promoters started in mid-log phase of growth and reached an apparent maximum in late exponential and early stationary phase of growth respectively (Fig. 4). However, the level of β-galactosidase activity was up to four times higher in the *oocG* promoter fusion strain (Fig. 4). Quantitative real-time PCR (qPCR) analyses were used to validate these transcriptional fusion experiments and showed that the transcript levels of *oocG-C* and *oocJ-W* were higher at stationary phase that in mid-exponential growth phase (Fig. S7).

### Quorum sensing-mediated regulation of oocydin A production is strain dependent

The highest levels of transcription of the *ooc* gene cluster occurred at higher cell densities suggesting that expression could be under QS control. Analysis of the genome sequence of *S. plymuthica* A153 revealed a candidate QS locus, SptIR and two orphan LuxR encoding genes, *splR* and *spsR*, all highly homologous to the *luxI*- and *luxR*-type genes encoded by the three QS loci present in *S. plymuthica* G3 (Liu *et al*., 2011; Duan *et al*., 2012). However, we were unable to detect the production of QS signalling molecules in A153 using the biosensor strains *Chromobacterium violaceum* CV026 (McClean *et al*., 1997) and *Serratia* SP19 (Poulter *et al*., 2010) (not shown). We therefore constructed mutants defective in the *sptI*, *sptR*, *splR* and *spsR* genes, and their phenotypic characterization demonstrated that, under the tested conditions, an AHL-based QS system does not seem to play a role in the production of oocydin A in A153 (Fig. S8).

**Fig. 5:**
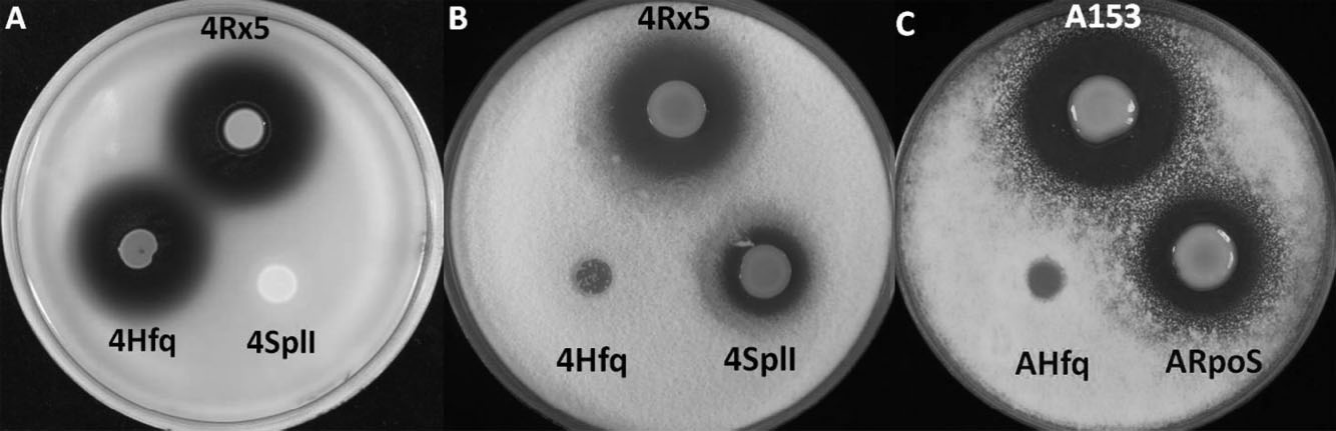
Quorum sensing, the chaperone Hfq and the sigma factor RpoS regulate the biosynthesis of oocydin A in *Serratia plymuthica* strains.A. Detection of AHLs produced by *S. plymuthica* 4Rx5 strains using the biosensor *Chromobacterium violaceum* CV026. AHL production is indicated by the production of purple haloes around the bacterial colonies.B and C. Bioactivities against *Verticillium dahliae* of *S. plymuthica* strain 4Rx5 (B), A153 (C) and derivative strains*.* The bioassays were repeated at least three times, and representative results are shown. CV026 and *V. dahliae* pictures were taken after 48 and 96 h of incubation at 25°C respectively. Bioactivities against the oomycete *Pythium ultimum* are shown in Figs S11 and S13. Mutants were complemented by *in trans* expression of the genes using pQE-80L-based vectors (Figs S11 and S13).

**Fig. 6:**
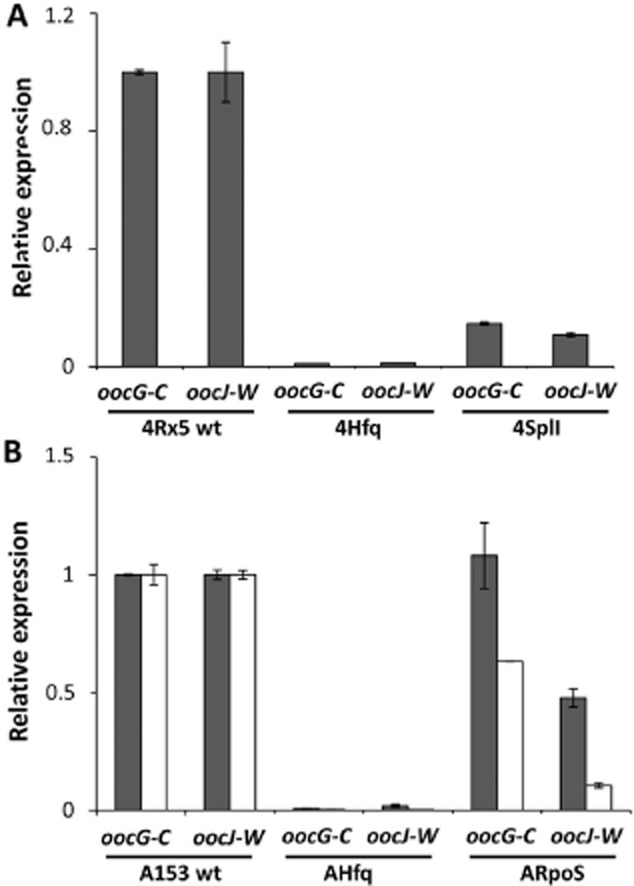
Impact of quorum sensing, RpoS and Hfq on *ooc* transcript levels measured by qPCR. Measurement of *oocG-C* and *oocJ-W* transcript levels in *Serratia plymuthica* 4Rx5 (A), *Serratia plymuthica* A153 (B) and their derivative strains. The values showed the average expression at early (grey) and late (white) stationary phase of growth relative to wild type expression. Arrows in Fig. 4 and Fig. S10 indicate time points when the samples for qPCR were taken. Data are the mean and standard deviation of three biological replicates.

Unlike A153, the oocydin A-producing strain *S. plymuthica* 4Rx5 produces QS signalling molecules that could be detected using the biosensor CV026 (Figs 5A and S10C). During the analysis of the genome of 4Rx5 (M. A. Matilla and G. P. C. Salmond, unpublished), we identified a QS locus homologous to the SplIR locus of *S. plymuthica* G3 (Liu *et al*., 2011). To investigate whether the production of oocydin A was regulated by QS in 4Rx5, we constructed an in frame deletion mutant defective in the AHL synthase gene *splI*. The resulting mutant did not produce QS signalling molecules (Fig. 5A) and showed reduced antifungal (Fig. 5B) and anti-oomycete (Fig. S9) properties – phenotypes that were complemented by the expression of *splI in trans* (Fig. S11A and B). qPCR analyses showed that *oocG-C* and *oocJ-W* transcripts levels in the *splI* strain were reduced to 14.6% and 10.8% of those of the wild type strain respectively (Fig. 6A).

Other oocydin A producers such as *S. marcescens* MSU97; *D. solani* strains MK10, MK16 and IPO 2222; and *Dickeya* sp. NCPPB 3274 also synthesize QS signalling molecules (Figs 1C and S12). To investigate the role of QS in the production of the bioactive macrolide in these strains, we used a quorum quenching approach in which, through the heterologous expression of the lactonase AiiA of *Bacillus* A24, the signalling molecules were enzymatically degraded (Fig. S12A). However, after the depletion of the AHLs, the bioactive properties of MSU97, MK10, MK16, IPO 2222 and NCPPB 3274 remained unaltered suggesting that oocydin A production in these five strains is not regulated by a AHL-based QS system (Fig. S12).

### RpoS differentially regulates the expression of the *ooc* transcriptional units

The higher *ooc* transcript levels in stationary phase of growth also encouraged us to investigate whether the stationary phase sigma factor, RpoS, could be regulating the expression of the *ooc* gene cluster. To test this hypothesis, we constructed a *rpoS*-deficient mutant of A153. The resulting mutant showed reduced antifungal (Fig. 5C) and anti-oomycete (Fig. S13B) properties reflecting significantly reduced levels of oocydin A produced by this strain. This decreased bioactivity was fully restored when complemented by the expression of *rpoS in trans* (Fig. S13). We assessed the effect of RpoS on the transcription of *oocG-C* and *oocJ-W*. Whereas the transcription of *oocG-C* remained unaltered in an *rpoS* mutant, the transcription of *oocJ-W* was reduced by around 50% throughout growth in the *rpoS*-deficient strain (Fig. 4). qPCR analyses demonstrated that the transcripts levels of *oocG-C* were unaltered in the *rpoS* mutant during early stationary growth, although slightly reduced when compared with the wild type strain during late stationary growth (Fig. 6B). As expected, the transcript levels of *oocJ-W* were reduced 2.1 ± 0.2 and 9.4 ± 0.9 times, respectively, during early and late stationary growth in a *rpoS*-deficient background (Fig. 6B). These results indicate that RpoS regulates the *oocJ-W* operon but not the *oocG-C* operon suggesting that different regulatory pathways independently modulate expression of the two *ooc* transcriptional units.

### The RNA-binding protein Hfq positively regulates the expression of the *ooc* gene cluster

It is well known that Hfq is required for *rpoS* translation in several enterobacterial strains by releasing transcript self-repression (Vogel and Luisi, 2011; Soper *et al*., 2010). In *Escherichia coli*, to release this self-repression, Hfq binds to an (AAN)_4_ motif found in the 5′untranslated region (UTR) of the *rpoS* transcript (Vogel and Luisi, 2011; Soper *et al*., 2010). We found that this (AAN)_4_ motif is highly conserved in the *rpoS* leader sequence within the *Serratia* genus, including the oocydin A-producing strains *S. plymuthica* A153, 4Rx5 and 4Rx13 and *S. marcescens* MSU97 (Fig. S14). To investigate whether Hfq is involved in the regulation of the biosynthesis of oocydin A, we constructed and characterized *hfq* deletion mutants of A153 and 4Rx5. In both strains, the deletion of *hfq* resulted in the complete loss of antifungal (Fig. 5) and anti-oomycete properties (Figs S9 and S13) indicating that production of oocydin A was abolished. These mutant phenotypes were complemented by the expression of *hfq in trans* (Figs S11 and S13). Although both *hfq* deletion mutants showed a slightly reduced growth rate and reached stationary phase at lower optical density, colony counts throughout growth showed no differences when compared with the A153 and 4Rx5 wild types (Fig. S10). β-Galactosidase assays showed that, whereas the transcription of *oocG-C* in A153 was reduced to around 50% of the wild type levels in a *hfq*-deficient strain, the transcription of *oocJ-W* was abolished (Fig. 4) again suggesting differential regulation of the two *ooc* transcriptional units. qPCR analyses of A153 confirmed that the transcript levels of *oocG-C* and *oocJ-W* were reduced by 99% in late stationary phase in A153 Δ*hfq* (Fig. 6B). Examination of the transcript levels of *oocG-C* and *oocJ-W* in *S. plymuthica* 4Rx5 confirmed that both were also drastically decreased in a Δ*hfq* background (Fig. 6A).

## Discussion

Although the first *trans*-AT PKS member was identified in the early 1990s (Scotti *et al*., 1993), it was almost a decade later that the first polyketide was associated with a *trans*-AT PKS gene cluster (Piel, 2002). Since then, an increasing number of polyketides have been recognized to be products of *trans*-AT PKS (Piel, 2010; Till and Race, 2014). The applications of these natural products are diverse and some have proved to be important from a pharmacological perspective (Hertweck, 2009; Piel, 2010). The haterumalide oocydin A is an example of a bioactive product of a *trans*-AT PKS and it has potential agricultural and pharmacological utility. Although the chemical synthesis of oocydin A has been achieved (Kigoshi and Hayakawa, 2007), overall yield is very low (Ueda *et al*., 2009). Consequently, the results presented in this study could lay the groundwork for more efficient production of novel oocydins with improved pharmacological, chemotherapeutic and agricultural properties.

Synthetic biology strategies are being used for the development of functionally optimized polyketides, and ATs are the main target for the re-engineering of *trans*-AT PKSs (Koryakina *et al*., 2013; Till and Race, 2014). However, although considerable progress has been made at biochemical (Lopanik *et al*., 2008; Musiol *et al*., 2011; 2013[Bibr b41],[Bibr b42]; Jensen *et al*., 2012) and structural levels (Cuskin *et al*., 2011; Wong *et al*., 2011), knowledge about the mechanisms of these enzymes is still limited. Particular attention is now being given to *trans*-ATs present in PKS biosynthetic clusters containing two or more free-standing ATs – including pederin (Piel, 2002), bacillaene (Chen *et al*., 2006), kirromycin (Weber *et al*., 2008), etnangien (Menche *et al*., 2008) and rhizopodin (Pistorius and Müller, 2012). In the *ooc* gene cluster, the domains OocV-AT1 and OocW-AT3 were predicted to be malonyl-CoA-specific ATs (Matilla *et al*., 2012). However, the role of OocV-AT2 in the biosynthesis of oocydin A remained elusive. Phylogenetic analyses showed that OocV-AT2 belongs to a clade that includes BryP-AT_2_ (41% identical, 62% similar), PedC (32% identical, 51% similar) and KirCl-AT_1_ (30% identical, 52% similar) (Fig. S15). Recently, it has been shown that PedC (Jensen *et al*., 2012) and KirCl-AT_1_ (Musiol *et al*., 2013) have no AT activity and that they may be acting as PKS proofreading factors to release stalled biosynthetic intermediates (Jensen *et al*., 2012). Importantly, although OocV-AT2 itself was not sufficient to restore oocydin A production in the OocVW strain, both OocV-AT1 and OocV-AT2 domains were required to fully complement to wild type levels of oocydin A (Fig. 3B). This result suggests that OocV-AT2 may be involved in catalysing the transfer of extender units preferentially to specific ACP(s), as described previously for other *trans-*ATs (Musiol *et al*., 2011; 2013). In accordance with this, and in contrast to PedC and KirCl-AT_1_, the domains OocV-AT2 and BryP-AT_2_ possess the conserved GHS*X*G motif and the domain BryP-AT_2_ has been shown to have AT activity (Lopanik *et al*., 2008).

The evolution of *trans*-AT PKS gene clusters remains an interesting area of research. It has been suggested that they mainly evolved through horizontal gene transfer (HGT) and assembly of domains between bacteria (Nguyen *et al*., 2008). Given the presence of phage-related genes bordering the *ooc* gene cluster in some oocydin A-producing strains, HGT was also suggested as the route to dissemination of the *ooc* gene cluster between the producing strains (Matilla *et al*., 2012). Consistent with this idea, we recently showed the mobilization of the complete *ooc* gene cluster at high frequencies by phage-mediated transduction (Matilla and Salmond, 2014) and the transduction of *ooc* mutations between several *D. solani* strains (Matilla and Salmond, 2014; Matilla *et al*., 2014). Further supporting this HGT hypothesis, in this study, we have shown that the *ooc* gene cluster is present in phylogenetically distant *Dickeya* strains (Naushad *et al*., 2014) and in several rhizobacterial strains isolated from the same crop (Berg *et al*., 2002).

The regulation of *trans*-AT polyketide synthesis is a largely unexplored area and, perhaps unexpectedly, no putative regulatory protein is encoded in the *ooc* gene cluster. In this study, we investigated, for the first time, the regulation of oocydin A biosynthesis. Under laboratory conditions, the biosynthesis of bacterial secondary metabolites is often growth phase dependent (Williamson *et al*., 2010; Wilf and Salmond, 2012; Liu *et al*., 2013) and expression of the *ooc* gene cluster is similarly enhanced at late stages of growth. Although we found no evidence that an AHL-based QS system regulates oocydin A biosynthesis in strains belonging to *Dickeya* genus, we demonstrated that the production of oocydin A is under QS control in *S. plymuthica* 4Rx5. Quorum sensing is involved in the regulation of various secondary metabolites made by some strains of *Serratia* (Thomson *et al*., 2000; Van Houdt *et al*., 2007a; Williamson *et al*., 2010). Furthermore, the role of QS in the biocontrol properties of several *S. plymuthica* strains, including the production of antifungal and antibacterial compounds, also has been established (Liu *et al*., 2007; 2011[Bibr b30],[Bibr b31]; Van Houdt *et al*., 2007b; Müller *et al*., 2009). The sequence of the 4Rx5 AHL synthase SplI is 99% identical to the corresponding synthases in the *S. plymuthica* strains HRO-C48 (Liu *et al*., 2007), RVH1 (Van Houdt *et al*., 2007b) and G3 (Liu *et al*., 2011). In these three strains, SplI is mainly responsible of producing *N*-3-oxo-hexanoyl homoserine lactone indicating that this signalling molecules may be the main AHL produced by 4Rx5.

The stationary sigma factor RpoS (Battesti *et al*., 2011) regulates, directly or indirectly, around 10% of *E. coli* genes (Weber *et al*., 2005). However, the role of RpoS in the regulation of bacterial biocontrol properties has been investigated mainly in *Pseudomonas* strains in which *rpoS* mutants can show increased (Sarniguet *et al*., 1995; Ge *et al*., 2007; Manuel *et al*., 2012) or decreased (Sarniguet *et al*., 1995; Park *et al*., 2011; Manuel *et al*., 2012) production of secondary metabolites – including antifungal compounds. In *S. plymuthica* A153, RpoS positively regulates the expression of the *ooc* gene cluster, preferentially activating the transcription of the *oocJ-W* transcriptional unit. RpoS was also shown to be required for the production of the antifungal compound pyrrolnitrin in *S. plymuthica* IC1270 (Ovadis *et al*., 2004). In contrast, an *rpoS* mutant of *Serratia* sp. ATCC 39006 showed increased expression of the prodigiosin and carbapenem biosynthetic clusters (Wilf and Salmond, 2012) demonstrating the complex and elastic regulatory networks that affect the synthesis of secondary metabolites within the *Serratia* genus.

The expression of *rpoS* is highly regulated and the RNA binding protein Hfq stimulates *rpoS* translation (Battesti *et al*., 2011; Vogel and Luisi, 2011; Wagner, 2013). However, in plant-associated bacteria, only a few recent studies have demonstrated the involvement of Hfq in their symbiotic efficiency (Gao *et al*., 2010) and virulence capabilities (Wilms *et al*., 2012; Zeng *et al*., 2013). In contrast, its importance in biocontrol bacteria is still poorly understood. Our results showed that Hfq positively regulates the expression of the *ooc* gene cluster in *S. plymuthica* strains A153 and 4Rx5. Hfq binding sites were found in the *rpoS* 5′UTR RNA of A153 and 4Rx5, consistent with Hfq regulation via RpoS. In fact, the loss of Hfq resulted in reduced *rpoS* transcript levels in A153 and 4Rx5 (Fig. S16) confirming the post-transcriptional regulation of *rpoS* by Hfq. Within the *Serratia* genus, Hfq has been shown to be involved in the regulation of the synthesis of pyrrolnitrin (Zhou *et al*., 2012), a carbapenem (Wilf *et al*., 2011) and a prodigiosin (Wilf *et al*., 2011). However, contrary to the results in this study, Hfq-mediated regulation of carbapenem and prodigiosin biosynthesis in *Serratia* sp. ATCC 39006 was shown to be independent of RpoS (Wilf and Salmond, 2012). Whether Hfq is directly regulating the expression of the *ooc* gene cluster is unknown, but *E. coli* Hfq has been shown to bind the 5′UTR RNA of the *carA* gene of the carbapenem gene cluster in *Serratia* sp. ATCC 39006 (Wilf *et al*., 2011).

## Concluding remarks

The unique architecture and unusual chemistry of various *trans*-AT PKS enzymes make these systems attractive targets for molecular re-engineering studies (Till and Race, 2014) – particularly in an era when discovery of new antimicrobial compounds is required. The results reported here highlight the complexity of the biosynthesis of the haterumalide, oocydin A, and the broad distribution of its biosynthetic gene cluster in *Serratia* and *Dickeya* strains. Mutagenesis and complementation analyses demonstrated the involvement of three Ooc *trans-*AT domains in the biosynthesis of the oocydin A. From this study, it is also clear that the biosynthesis of oocydin A is highly regulated and we also provide data supporting the view that different environmental and physiological signals may regulate the biosynthesis of oocydin A, in a strain-dependent fashion.

## Experimental procedures

### Bacterial strains, culture media and growth conditions

Bacterial strains used in this study are listed in Table S2. *Serratia*, *Dickeya*, *Pantoea*, *Xenorhabdus* and their derivative strains were routinely grown at 30°C, unless otherwise indicated, in Luria Broth (LB; 5 g yeast extract l^−1^, 10 g Bacto tryptone l^−1^ and 5 g NaCl l^−1^), Potato Dextrose (24 g potato dextrose broth l^−1^) or minimal medium [0.1%, w/v, (NH_4_)_2_SO_4_, 0.41 mM MgSO_4_, 0.2% (w/v) glucose, 40 mM K_2_HPO_4_, 14.7 mM KH_2_PO_4_, pH 6.9–7.1]. *Escherichia coli* strains were grown at 37°C in LB. *Escherichia coli* DH5α was used as a host for gene cloning. When appropriate, antibiotics were used at the following final concentrations (in microgram per millilitre): ampicillin, 100; chloramphenicol, 25; kanamycin, 25 (*E. coli* strains) and 50 (*Serratia* strains); streptomycin, 50; tetracycline, 10 (*E. coli* strains) and 20 (*Serratia* strains). Sucrose was added to a final concentration of 10% (w/v) when required to select derivatives that had undergone a second cross-over event during marker exchange mutagenesis. Bacterial growth (OD_600 nm_) was measured on a Unicam Helios spectrophotometer at 600 nm, 1 cm path length.

### *In vitro* nucleic acid techniques and bioinformatics analyses

Plasmid DNA was isolated using the Anachem Keyprep plasmid kit. For DNA digestion, the manufacturer's instructions were followed (New England Biolabs and Fermentas). Separated DNA fragments were recovered from agarose using the Anachem gel recovery kit. Ligation reactions and total DNA extraction were performed as previously described (Sambrook *et al*., 1989). Competent cells were prepared using calcium chloride and transformations were performed by standard protocols (Sambrook *et al*., 1989). Phusion high-fidelity DNA polymerase (New England Biolabs) was used in the amplification of PCR fragments for cloning. PCR reactions were purified using the Anachem PCR Clean-up kit. Sequences of the PCR fragments were verified in order to discard amplicons containing mutations. Routine DNA sequencing was carried out at the University of Cambridge DNA Sequencing Facility on an Applied Biosystems 3730xl DNA Analyser. Genome comparison analyses were performed employing wgVISTA online tool (Frazer *et al*., 2004). Blast analyses were made for the functional gene assignment. Protein domain organization was identified using the NCBI conserved domains database. Multiple sequence alignments were carried out with ClustalW2 (European Bioinformatics Institute).

### Construction of strains and plasmids

The procedures for the generation of bacterial strains and plasmids are described in Appendix S1.

### Phenotypic assays

Antagonistic activities of bacterial strains against the fast-growing plant pathogenic oomycete, *P. ultimum*, and the fungus, *V. dahliae*, were assayed as described previously (Matilla *et al*., 2012). In all cases, 5 μl of overnight cultures of the selected strains were spotted on the surface of the bioassay plates. Production of *N*-AHLs was detected using the *C. violaceum* CV026 (McClean *et al*., 1997) and *Serratia* SP19 (Poulter *et al*., 2010) bacterial biosensor strains. Quorum quenching assays by the heterologous expression of the AiiA acyl-homoserine lactonase from *Bacillus* sp. A24 were performed as described previously (Liu *et al*., 2011). Inactivation of the AHLs produced by the tested strains was evaluated by using CV026 and SP19 biosensor strains.

### Genetic complementation assays

Complementation of mutations was carried out by the introduction of a wild type copy of the corresponding mutated gene *in trans* on a plasmid. For the complementation assays, LB agar (LBA) containing the appropriate antibiotic (to maintain the plasmid) and isopropyl-β-D-thiogalactopyranoside (IPTG) at 0.1 or 1 mM was added to holes punched in *P. ultimum*, *V. dahliae*, SP19 or CV026 bioassay plates. Then, 5 μl of overnight cultures of the selected strains were spotted on the surface of the LBA containing the antibiotic and IPTG and incubated for 2–5 days at 25°C or 30°C.

### Generalized transduction

The generalized transducing Viunalikevirus, ΦMAM1, was used for transduction of chromosomal mutations, as described previously (Matilla and Salmond, 2014).

### Transcriptional fusion assays

Expression of the *lacZ* reporter gene was performed as described previously (Ramsay, 2013) using the fluorogenic substrate 4-methylumbelliferyl β-D-galactoside (Melford Cat No. M1095) at a final concentration of 0.125 mg ml^−1^. Samples were measured in a SpectraMax Gemini XPS fluorescence microplate reader (Molecular Devices) using the following settings: excitation 360 nm, emission 450 nm, cut-off 435 nm, reading every 30 s for 20 min at 37°C. β-Galactosidase activity was expressed as relative fluorescent units s^–1^ and normalize to the OD_600 nm_ of the corresponding sample. All the transcriptional fusion assays were performed using *S. plymuthica* A153 LacZ (wild type) or mutants derived from LacZ.

### RNA extraction and qPCR

RNA was extracted from exponential and stationary phase cultures grown in LB medium (see Figs 4 and S10) using an RNeasy mini kit (Qiagen) according to the manufacturer's instructions. RNA concentration was determined spectrophotometrically and RNA integrity was assessed by agarose gel electrophoresis. Genomic DNA contamination was eliminated by treating total RNA with Turbo DNA free (Ambion). The synthesis of cDNA was performed using random hexamers (GE healthcare) and SuperScript II reverse transcriptase (Invitrogen) in a 30 μl reaction with 2.0 μg of total RNA and incubation at 42°C for 2 h. A negative control reaction was also performed, omitting the reverse transcriptase enzyme. qPCRs were performed as described previously (Burr *et al*., 2006) using primers specific for *oocE*, *oocJ* and *rpoS* (Table S4). qPCR amplifications were performed using an ABI PRISM 7000 sequence detection system. To confirm absence of contaminating genomic DNA, control PCRs were carried out using no RT cDNA samples as templates. Melting curve analyses were conducted to ensure amplification of a single product. The relative gene expression was calculated using the critical threshold (ΔΔCt) method (Pfaffl, 2001) and using 16S rRNA as the internal control to normalize the data.

## Supporting Information

**Fig. S1.** Structure of oocydin A. **Fig. S2.** The oocydin A gene cluster is widespread within the *Dickeya* genus. The alignments represent the percentage of DNA homology between the oocydin A (*ooc*) gene cluster of *Dickeya paradisiaca* Ech703 and the those of *Dickeya solani* strains MK10 (A), MK16 (B), IPO 2222 (C), GBBC 2040 (D), 3337 (E) and D_s0432-1 (F), *Dickeya chrysanthemi* NCPPB 402 (G), *Dickeya* sp. CSL RW240 (H), *Dickeya* sp. NCPPB 3274 (I), *Dickeya dianthicola* strains NCPPB 453 (J), GBBC 2039 (K), NCPPB 3534 (L), IPO 980 (M) and *Dickeya paradisiaca* NCPPB 2511 (N). Alignments were performed using wgVISTA (Frazer *et al*., 2004). **Fig. S3.** The oocydin A gene cluster is present in several enterobacterial strains isolated from the rhizosphere of agronomically important crops. A. Representation of the *ooc* gene cluster in *Serratia marcescens* MSU97. Lines labelled 1–24 above the gene cluster represent the regions amplified in the PCR shown in B. Numbers below the arrows represent the intergenic distance between contiguous genes, with negative numbers indicate overlapping genes. Polyketide synthase encoding genes are shown in green. Genes flanking the *ooc* gene cluster are shown in red. B. Analysis by PCR using primers designed to span the intergenic region between two adjacent genes of the *ooc* gene cluster in MSU97. C. Antifungal activities against *Verticillium dahliae*. The bioassays were repeated at least three times, and representative results are shown. *Verticillium dahliae* pictures were taken after 96 h of incubation at 25°C respectively. **Fig. S4.** Schematic representation of the oocydin A gene cluster in *Serratia plymuthica* 4Rx5. Numbers below the arrows represent the intergenic distance between contiguous genes, with negative numbers indicate overlapping genes. **Fig. S5.** Anti-oomycete activities of *Serratia plymuthica* A153 and derivative strains with mutations in the *ooc* gene cluster. Bioactivities of *S. plymuthica* A153 strains against *Pythium ultimum* are shown. The bioassays were repeated at least three times, and representative results are shown. Pictures were taken after 48 h of incubation at 25°C. Complementation assays are shown in Fig. S6. **Fig. S6.** Genetic complementation of *Serratia plymuthica* A153 mutants mapping to the *ooc* gene cluster. Bioactivities against *Verticillium dahliae* (A) and *Pythium ultimum* (B) are shown. *Pythium ultimum* inoculation points are indicated with a black dot. *Pythium ultimum* and *V. dahliae* pictures were taken after 48 and 96 h of incubation at 25°C respectively. Induction of the expression of the Ooc proteins was done by addition of 1 mM of IPTG. **Fig. S7.** Impact of growth phase on *ooc* transcript levels measured by qPCR. Measurement of *oocG-C* and *oocJ-W* transcript levels in *Serratia plymuthica* A153 (A) and *S. plymuthica* 4Rx5 (B) along the growth curve. The values showed the average expression relative to the expression at mid logarithmic phase of growth (OD600 ∼0.8). Arrows in Fig. 4 and Fig. S10 indicate time points when the samples for qPCR were taken. Data are the mean and standard deviation of three biological replicates. **Fig. S8.** An AHL-based quorum sensing system does not regulate the biosynthesis of oocydin A in *Serratia plymuthica* A153. Antifungal activities against *Verticillium dahliae* of *S. plymuthica* A153 strains are shown. The bioassays were repeated at least three times, and representative results are shown. Pictures were taken after 96 h of incubation at 25°C. **Fig. S9.** Bioactivities of *Serratia plymuthica* 4Rx5 strains against *Pythium ultimum*. The inoculation points of *Pythium ultimum* and 4Hfq are indicated with a black or red dot respectively. The bioassays were repeated three times, and representative results are shown. Pictures were taken after 48 and 72 h of incubation at 25°C respectively. **Fig. S10.** Growth and quorum sensing signalling molecule production in *Serratia plymuthica* strains. Turbidity (filled symbols) and colony-forming units per millilitre (cfu ml^–1^; open symbols) of *Serratia plymuthica* A153 (A), *S. plymuthica* 4Rx5 (B) and their derivative strains, in LB medium at 25°C are shown. Data are the mean and standard deviation of three biological replicates. Arrows, time points where samples for quantitative RT-PCR were taken. (C) Detection of AHLs in *S. plymuthica* 4Rx5 filter-sterilized supernatants throughout growth by biosensor strain CV026. Quorum sensing pictures were taken after 24 h of incubation at 25°C. **Fig. S11.** Complementation assays of *Serratia plymuthica* 4Rx5 strains 4SplI and 4Hfq. Expression of *splI in trans* in the strain 4SplI restored AHLs production (A) and complemented the antifungal activity against *Verticillium dahliae* (B). Expression of *hfq in trans* complemented the antifungal (C) and anti-oomycete (D) activities in the strain 4Hfq. In D, the inoculation points of *Pythium ultimum* and 4Hfq are indicated with a black or red dot respectively. Induction of the expression of the Hfq and SplI proteins was done by addition of 0.1 mM of IPTG. CV026, *Pythium ultimum* and *V. dahliae* pictures were taken after 48, 48 and 96 h of incubation at 25°C respectively. **Fig. S12.** An AHLs-based quorum sensing is not involved in the regulation of oocydin A in several enterobacteria strains. Effect of *aiiA* expression on the production of AHLs detected by using the biosensor strains SP19 (12) or CV026 (11) (A). Their bioactivities against the oomycete *Pythium ultimum* are also shown in B. The bioassays were repeated at least three times, and representative results are shown. CV026 and anti-oomycete pictures were taken after 48 h of incubation at 25°C. SP19 pictures were taken after 48 h at 30°C. **Fig. S13.** Complementation assays of *Serratia plymuthica* A153 strains RpoS and AHfq. Expression of *rpoS in trans* in the strain ARpoS complemented the antifungal (A) and anti-oomycete (B) activities against *Verticillium dahliae* and *Pythium ultimum* respectively. *In trans* expression of *hfq* complemented the antifungal (C) and antioomycete (D) activities in the strain AHfq. In D, the inoculation points of *Pythium ultimum* and AHfq are indicated with a black or red dot respectively. Induction of the expression of the RpoS and SplI proteins was done by addition of 0.1 mM of IPTG. *Pythium ultimum* and *V. dahliae* pictures were taken after 48 and 96 h of incubation at 25°C respectively. **Fig. S14.** Multiple alignments of the untranslated regions of the *rpoS* leader from different *Serratia* strains. The (AAN)4 motif and the A6 element, essential for the Hfq binding (Soper *et al*., 2010), are highlighted in blue and yellow respectively. Start codon (grey) and Shine-Dalgarno (underlined) sequences are also shown. Multiple sequence alignments were carried out with ClustalW2 (European Bioinformatics Institute). SM39, *Serratia marcescens* SM39; Db11, *Serratia marcescens* Db11; WW4, *Serratia marcescens* WW4; A153, *Serratia plymuthica* A153; 4Rx5, *Serratia plymuthica* 4Rx5; 4Rx13, *Serratia plymuthica* 4Rx13; S13, *Serratia* sp. S13; AS13, *Serratia plymuthica* AS13; AS12, *Serratia plymuthica* AS12; AS9, *Serratia plymuthica* AS9; ATCC_27592, *Serratia liquefaciens* ATCC_27592; 598, *Serratia proteomaculans* 598; FGI94, *Serratia marcescens* FGI94; V4, *Serratia plymuthica* V4; RB-25, *Serratia fonticola* RB-25. **Fig. S15.** Phylogenetic tree of the *trans*-acyltransferase (AT) domains from several *trans*-AT PKS gene clusters. Multiple sequence alignments were carried with ClustalW (European Bioinformatics Institute). The phylogenetic trees were generated with the MEGA software v6 (Tamura *et al*., 2013) using the neighbour-joining method with *P* distance values*.* Bootstrap values (expressed as percentages of 500 replicates) are shown at branch points. **Fig. S16.** Impact of Hfq on *rpoS* transcript levels measured by qPCR. Measurement of *rpoS* transcript levels in *Serratia plymuthica* strains A153 and 4Rx5. The values showed the average expression at stationary phase of growth relative to the wild type expression. Samples for qPCR were taken after growing A153 and 4Rx5 strains in LB medium at 25°C for 6 and 8 h respectively (see Fig. S10). Data are the mean and standard deviation of three biological replicates. **Table S1.** Identity at DNA level of the oocydin A gene cluster between *Dickeya* strains. **Table S2.** Bacteria, oomycete, fungi and phages used in this study. **Table S3.** Plasmids used in this study. **Table S4.** Oligonucleotides used in this study. **Appendix S1.** Extended experimental procedures: *Construction of strains and plasmids*.
