# Supplementary material for: Biosynthesis of the antifungal haterumalide, oocydin A, in Serratia, and its regulation by quorum sensing, RpoS and Hfq
Source: Environ Microbiol. 2015 Apr 8;17(8):2993–3008. doi: 10.1111/1462-2920.12839 (PMC4552970; doi:10.1111/1462-2920.12839)
Supplement: Supplementary file 1 [file emi0017-2993-sd1.pdf]

Supporting information

Biosynthesis of the antifungal haterumalide, oocydin A, in *Serratia,* and its regulation by quorum sensing, RpoS and Hfq.

Miguel A. Matilla[a], Finian J. Leeper[b], George P.C. Salmond[a]*

[a]Department of Biochemistry, University of Cambridge, Tennis Court Road, Cambridge, UK, CB2 1QW.

[b]Department of Chemistry, University of Cambridge, Lensfield Road, Cambridge, UK, CB2 1EW.

Running title: *Biosynthesis and regulation of oocydin A*

*Address correspondence to George P.C. Salmond, Department of Biochemistry, University of Cambridge, Tennis Court Road, Cambridge, UK, CB2 1QW. Tel: +44 (0)1223 333650; Fax: +44 (0)1223 766108; E-mail: gpcs2@cam.ac.uk

Supplementary Table S1: Identity at DNA level of the oocydin A gene cluster between *Dickeya* strains.

DNA homology (%)	MK10	MK16	IPO 2222	GBBC 2040	*3337*	D s0432-1	NCPPB 402	CSL RW240	NCPPB 3274	NCPPB 453	GBBC 2039	IPO 980	NCPPB 2511	Ech703
***Dickeya solani* MK10**	X	100	99.9	99.9	100	100	80.5	89	81.5	89.9	89.1	91.1	85.6	85.6
***D. solani* MK16**	100	X	99.9	99.9	100	100	80.5	89	81.5	89.9	89.1	91.1	85.6	85.6
***D. solani* IPO 2222**	99.9	99.9	X	99.9	99.9	99.9	80.4	88.9	81.4	89.8	89	90	85.5	85.6
***D. solani* GBBC 2040**	99.9	99.9	99.9	X	99.9	99.9	78.2	88.1	79.2	87.5	88.1	87.5	83.3	83.4
***D. solani* 3337**	100	100	99.9	99.9	X	100	80.5	89	81.5	89.9	89.1	90.1	85.6	85.6
***D. solani* D s0432-1**	100	100	99.9	99.9	100	X	80.5	89	81.5	89.9	89.1	91.1	85.6	85.6
***D. chrysanthemi* NCPPB 402**	80.5	80.5	80.4	78.2	80.5	80.5	X	79.4	94.5	80.4	79.4	80.3	78.8	78.9
***Dickeya* sp. CSL RW240**	89	89	88.9	88.1	89	89	79.4	X	80.2	97.8	100	98.2	84.2	84.2
***Dickeya* sp. NCPPB 3274**	81.5	81.5	81.4	79.2	81.5	81.5	94.5	80.2	X	81.2	80.3	81.2	79.7	79.7
***D. dianthicola* NCPPB 453**	89.9	89.9	89.8	87.5	89.9	89.9	80.4	97.8	81.2	X	97.8	98.7	85.2	85.2
***D. dianthicola* GBBC 2039**	89.1	89.1	89	88.1	89.1	89.1	79.4	100	80.3	97.8	X	98.2	84.2	84.3
***D. dianthicola* IPO 980**	91.1	90.1	90	87.5	90.1	91.1	80.3	98.2	81.2	98.7	98.2	X	85.2	85.2
***D. paradisiaca* NCPPB 2511**	85.6	85.6	85.5	83.3	85.6	85.6	78.8	84.2	79.7	85.2	84.2	85.2	X	100
***D. paradisiaca* Ech703**	85.6	85.6	85.6	83.4	85.6	85.6	78.9	84.2	79.7	85.2	84.3	85.2	100	X

Supplementary Table S2. Bacteria, oomycete, fungi and phages used in this study

Bacteria/fungi/oomycete/ phage/	Genotype or relevant characteristic[a]	Reference or source
Escherichia coli DH5α	*supE44 lacU169*(*Ø80lacZΔ M15*) *hsdR17* ($r_K^- m_K^-$) *recA1 endA1 gyrA96 thi-1 relA1*	Woodcock *et al*., (1989)
E. coli CC118λpir	*araD*, Δ(*ara*, *leu*), Δ*lacZ*74, *pho*A20, *galK*, *thi-1*, *rspE*, *rpoB*, *argE*, *recA1*, λ*pir*	Herrero *et al*., (1990)
E. coli HH26	Mobilizing strain for conjugal transfer	Kaniga *et al*., (1991)
E. coli β2163	F^- RP4-2-Tc::Mu D*dapA*::(*erm-pir*), Km^R Em^R	Demarre *et al*., (2005)
Serratia plymuthica A153	Wild type, rhizosphere isolate	Hökeberg *et al*., (1997)
LacZ	A153 Δ*lacZ* (1470 bp Δ)	This study
OOA	A153 in-frame Δ*oocA* (711 bp Δ)	This study
OOB	A153 in-frame Δ*oocB* (474 bp Δ)	This study
OOE	A153 in-frame Δ*oocE* (1089 bp Δ)	This study
OOK	A153 in-frame Δ*oocK* (732 bp Δ)	This study
OOM	A153 in-frame Δ*oocM* (894 bp Δ)	This study
OcO	A153 in-frame Δ*oocO* (210 bp Δ)	This study
OOP	A153 in-frame Δ*oocP* (903 bp Δ)	This study
OOQ	A153 in-frame Δ*oocQ* (258 bp Δ)	This study
OOT	A153 in-frame Δ*oocT* (681 bp Δ)	This study
OOU	A153 in-frame Δ*oocU* (1185 bp Δ)	This study
OOV-AT1	A153 in-frame deletion of AT1 domain encoding region of *oocV* (933 bp Δ)	This study
OOV-AT2	A153 in-frame deletion of AT2 domain encoding region of *oocV* (843 bp Δ)	This study
OOV	A153 in-frame Δ*oocV* (1758 bp Δ)	This study
OOW	A153 *oocW*::Km, Km^R	This study
AT1W	A153 in-frame deletion of AT1 domain encoding region of *oocV* (933 bp Δ), *oocW*::Km, Km^R	This study
OocVW	A153 in-frame Δ*oocV-oocW* (2924 bp Δ)	This study
OOJ-ACP	A153 with G*X*DS motif of the ACP_L domain mutated to GV**GA**	This study
OOS-TE	A153 with G*X*S*X*G motif of the TE domain mutated to GY**A**MG	This study
OOS-C	A153 with HH*XXX*DG motif of the C domain mutated to HHFHA**A**G	This study
MMnO10	A153 transposon mutant *oocJ*::Tn-KRCPN1, Km^R	Matilla *et al*., (2012)
MMnO14	A153 transposon mutant *oocC*::Tn-KRCPN1, Km^R	Matilla *et al*., (2012)
MMnO15	A153 transposon mutant *oocU*::Tn-KRCPN1, Km^R	Matilla *et al*., (2012)
ARpoS	A153 *rpoS*::Km, Km^R	This study
AHfq	A153 Δ*hfq*::Km (252 bp Δ), Km^R	This study
RpoSL	Δ*lacZ, rpoS*::Km; generated by transduction using ϕMAM1, Km^R	This study
AHfqL	Δ*lacZ* , Δ*hfq*::Km; generated by transduction using ϕMAM1, Km^R	This study
ASptI	A153 in-frame *sptI* (483 bp Δ)	Matilla and Salmond, unpublished

ASptR	A153 *sptR*::Km, Km^R	Matilla and Salmond, unpublished
ASplR	A153 *splR*::Km, Km^R	Matilla and Salmond, unpublished
ASpsR	A153 *spsR*::Km, Km^R	Matilla and Salmond, unpublished
S. plymuthica 4Rx5	Wild type, rhizosphere isolate	Berg *et al*., (2002)
4Hfq	4Rx5 Δ*hfq*::Km (252 bp Δ), Km^R	This study
4SplI	4Rx5 in-frame *splI* (522 bp Δ)	This study
Serratia marcescens MSU97	Wild type, plant epiphyte, pigmented	Strobel *et al*., (1999)
Serratia fonticola 3Rc3	Wild type, rhizosphere isolate	Berg *et al*., (2002)
Serratia proteamaculans 3Rc15	Wild type, rhizosphere isolate	Berg *et al*., (2002)
Xenorhabdus luminescens 3Rp5	Wild type, rhizosphere isolate	Berg *et al*., (2002)
Pantoea agglomerans 3Rc14	Wild type, rhizosphere isolate	Berg *et al*., (2002)
Dickeya paradisiaca Ech703	Wild type, plant pathogen	CP001654
D. solani MK10	Wild type, plant pathogen	Pritchard *et al*., (2013a)
D. solani MK16	Wild type, plant pathogen	Pritchard *et al*., (2013a)
D. solani IPO 2222	Wild type, plant pathogen	Pritchard *et al*., (2013a)
D. chrysanthemi NCPPB 402	Wild type, plant pathogen	Pritchard *et al*., (2013b)
D. dianthicola IPO 980	Wild type, plant pathogen	Pritchard *et al*., (2013a)
Dickeya sp. NCPPB 3274	Wild type, plant pathogen	Pritchard *et al*., (2013b)
Chromobacterium violaceum CV026	*cviI*::Tn5, AHLs bioassay strain	McClean *et al*., (1997)
Serratia SP19	*smaI*::mini-Tn5Sm/Sp, *pigX*::Tn-DS1028, *pigZ*::miniTn5lacZ1; AHLs bioassay strain; Sp^R, Cm^R, Km^R	Poulter *et al*., (2010)
**Fungi/oomycete strains**		
Pythium ultimum	Wild type, plant pathogen	C.A.Gilligan
Verticillium dahliae 5368	Wild type, plant pathogen	R. Cooper
**Phages**		
ϕMAM1	Generalized transducing phage for *S. plymuthica* A153	Matilla and Salmond (2014)

[a]Cm, Chloramphenicol; Km, kanamycin; Sm, streptomycin; Sp, spectinomycin; Tc, tetracycline; Em, erythromycin.

Supplementary Table S3. Plasmids used in this study.

Plasmid	Relevant characteristic[a]	Primers used for cloning	Source
pKNG101	Sm^r; *oriR6K mob sacBR*		Kaniga *et al*., (1991)
pUC18Not	Ap^r; identical to pUC18 but with two NotI sites flanking pUC18 polylinker		Herrero *et al*., (1990)
p34S-Km3	Km^r, Ap^r; *Km3* antibiotic cassette		Dennis and Zylstra (1998)
pNJ5000	Tc^r; Mobilizing plasmid used in marker exchange		Grinter (1983)
pTRB30	Km^r; pQE-80L (Qiagen) based expression vector, Ap^r resistance cassette replaced by Km^r. IPTG-inducible promoter, ColE1 origin.		T. Blower
pTA100	Sm^r, Sp^r; pQE-80L (Qiagen) based expression vector with Ap^r resistance cassette replaced by Sm/Sp. IPTG-inducible promoter, ColE1 origin.		Fineran *et al*.,(2009)
pMP220	Tc^r; *oriRK2 'lacZ*		Spaink *et al*., (1987)
pME6000	Tc^r; Broad-host-range cloning vector		Maurhofer *et al*., (1998)
pME6883	Tc^r ; pME6000 carrying the *aiiA* gene of *Bacillus* strain A24 under constitutive *plac* control		Reimmann *et al*., (2002)
pMAMV108	Ap^r; 2.7-kb EcoRI/HindIII PCR product containing a region of the *lacZ* gene of A153 inserted into the EcoRI/HindIII sites of pUC18Not	1,2	This study
pMAMV110	Ap^r; pMAMV108 with a 1.4-kb PstI deletion in the *lacZ* gene of A153		This study
pMAMV112	Sm^r; 1.3-kb NotI fragment of pMAMV110 was cloned at the same site in pKNG101		This study
pMAMV118	Ap^r; 1.3-kb PCR product containing a 711 bp in frame deletion of *oocA* of A153 inserted into the EcoRI/PstI sites of pUC18Not	3/4 and 5/6	This study
pMAMV122	Sm^r; 1.4-kb NotI fragment of pMAMV118 was cloned at the same site in pKNG101		This study
pMAMV125	Ap^r; 1.5-kb PCR product containing a 474 bp in frame deletion of *oocB* of A153 inserted into the EcoRI/HindIII sites of pUC18Not	7/8 and 9/10	This study
pMAMV133	Sm^r; 1.6-kb NotI fragment of pMAMV125 was cloned at the same site in pKNG101		This study
pMAMV68	Sm^r; 1.5-kb PCR product containing a 1089 bp in frame deletion of *oocE* of A153 inserted into the SalI/ApaI sites of pKNG101	11/12 and 13/14	This study
pMAMV64	Ap^r; 1.5-kb PCR product containing a 732 bp in frame deletion of *oocK* of A153 inserted into the EcoRI/PstI sites of pUC18Not	15/16 and 17/ 18	This study
pMAMV65	Sm^r; 1.6-kb NotI fragment of pMAMV64 was cloned at the same site in pKNG101		This study
pMAMV63	Ap^r; 1.1-kb PCR product containing a 894 bp in frame deletion of *oocM* of A153 inserted into the EcoRI/PstI sites of pUC18Not	19/20 and 21/22	This study
pMAMV66	Sm^r; 1.2-kb NotI fragment of pMAMV63 was cloned at the same site in pKNG101		This study
pMAMV119	Ap^r; 1.4-kb PCR product containing a 210 bp in frame deletion of *oocO* of A153 inserted into the EcoRI/PstI sites of pUC18Not	23/24 and 25/26	This study
pMAMV123	Sm^r; 1.5-kb NotI fragment of pMAMV119 was cloned at the same site in pKNG101		This study
pMAMV126	Ap^r; 1.4-kb PCR product containing a 903 bp in frame deletion of *oocP* of A153 inserted into the EcoRI/HindIII sites of pUC18Not	27/28 and 29/30	This study
pMAMV134	Sm^r; 1.5-kb NotI fragment of pMAMV126 was cloned at the same site in pKNG101		This study
pMAMV120	Ap^r; 1.2-kb PCR product containing a 258 bp in frame deletion of *oocQ* of A153 inserted into the EcoRI/PstI sites of pUC18Not	31/32 and 33/34	This study
pMAMV124	Sm^r; 1.3-kb NotI fragment of pMAMV120 was cloned at the same site in pKNG101		This study
pMAMV69	Ap^r; 1.3-kb PCR product containing a 681 bp in frame deletion of *oocT* of A153 inserted into the EcoRI/PstI sites of pUC18Not	35/36 and 37/ 38	This study
pMAMV71	Sm^r; 1.4-kb NotI fragment of pMAMV69 was cloned at the same site in pKNG101		This study
pMAMV67	Sm^r; 1.5-kb PCR product containing a 1185 bp in frame deletion of *oocU* of A153 inserted into the SalI/ApaI sites of pKNG101	39/40 and 41/ 42	This study

pMAMV82	Ap^r; 1.5-kb PCR product containing a 933 bp in frame deletion of the acyltransferase domain 1 (AT1) of *oocV* of A153 inserted into the EcoRI/PstI sites of pUC18Not	43/44 and 45/46	This study
pMAMV86	Sm^r; 1.5-kb NotI fragment of pMAMV82 was cloned at the same site in pKNG101		This study
pMAMV83	Ap^r; 1.7-kb PCR product containing a 843 bp in frame deletion of the acyltransferase domain 2 (AT2) of *oocV* of A153 inserted into the EcoRI/PstI sites of pUC18Not	47/48 and 49/50	This study
pMAMV87	Sm^r; 1.7-kb NotI fragment of pMAMV83 was cloned at the same site in pKNG101		This study
pMAMV85	Ap^r; 1.5-kb PCR product containing a 1758 bp in frame deletion of AT1 and AT2 of *oocV* of A153 inserted into the EcoRI/PstI sites of pUC18Not	43/44 and 50/51	This study
pMAMV89	Sm^r; 1.5-kb NotI fragment of pMAMV85 was cloned at the same site in pKNG101		This study
pMAMV81	Ap^r; 1.6-kb PCR product containing a region of *oocW* of A153 inserted into the EcoRI/PstI sites of pUC18Not	52,53	This study
pMAMV84	Ap^r, Km^r; 0.95 kb SmaI fragment containing *km3* cassette of p34S-Km3 was inserted into EcoRV site of *oocW* in pMAMV81		This study
pMAMV88	Sm^r; Km^r; 2.6-kb NotI fragment of pMAMV84 was cloned at the same site in pKNG101		This study
pMAMV178	Ap^r; 1.5-kb PCR product containing a 2924 bp in frame deletion of *oocV*-*oocW* of A153 inserted into the EcoRI/HindIII sites of pUC18Not	43/54 and 55/56	This study
pMAMV180	Sm^r; 1.5-kb NotI fragment of pMAMV178 was cloned at the same site in pKNG101		This study
pMAMV95	Ap^r; 1.5-kb PCR product with G*X***DS** motif of the ACP_L domain mutated to GV**GA** and inserted into the EcoRI/HindIII sites of pUC18Not	57/58 and 59/60	This study
pMAMV99	Sm^r; 1.5-kb NotI fragment of pMAMV95 was cloned at the same site in pKNG101		This study
pMAMV96	Ap^r; 1.5-kb PCR product with G*X***S***X*G motif of the TE domain mutated to GY**A**MG and inserted into the EcoRI/HindIII sites of pUC18Not	61/62 and 63/64	This study
pMAMV100	Sm^r; 1.5-kb NotI fragment of pMAMV96 was cloned at the same site in pKNG101		This study
pMAMV97	Ap^r; 1.5-kb PCR product with HH*XXX***D**G motif of the NRPS-Condensation domain mutated to HHFHA**A**G and inserted into the EcoRI/HindIII sites of pUC18Not	65/66 and 67/68	This study
pMAMV101	Sm^r; 1.5-kb NotI fragment of pMAMV97 was cloned at the same site in pKNG101		This study
pMAMV117	Ap^r; 1.4-kb PCR product containing a 252 bp deletion of *hfq* of A153 and 4Rx5 inserted into the EcoRI/SphI sites of pUC18Not	69/70 and 71/72	This study
pMAMV139	Ap^r, Km^r; 0.95 kb BamHI fragment containing *km3* cassette of p34S-Km3 was inserted into newly inserted BamHI site of *hfq* in pMAMV117		This study
pMAMV160	Sm^r, Km^r; 2.4-kb NotI fragment of pMAMV139 was cloned at the same site in pKNG101		This study
pMAMV109	Ap^r; 1.5-kb EcoRI/HindIII PCR product containing the *rpoS* gene of A153 was inserted into the EcoRI/HindIII sites of pUC18Not	73,74	This study
pMAMV111	Ap^r, Km^r; 0.95 kb BamHI fragment containing *km3* cassette of p34S-Km3 was inserted into BamHI site of *rpoS* in pMAMV109		This study
pMAMV113	Sm^r, Km^r; 2.5-kb NotI fragment of pMAMV111 was cloned at the same site in pKNG101		This study
pMAMV179	Ap^r; 1.4-kb PCR product containing a 522 bp deletion of *splI* of 4Rx5 was inserted into the EcoRI/HindIII sites of pUC18Not	75/76 and 77/78	This study
pMAMV181	Sm^r; 1.5-kb NotI fragment of pMAMV179 was cloned at the same site in pKNG101		This study
pOocC	Sm^r; *oocC* gene was cloned into the BamHI/HindIII sites of pTA100	79,80	This study
pOocE	Km^r; *oocE* gene was cloned into the BamHI/SalI sites of pTRB30	81,82	This study
pOocK	Km^r; *oocK* gene was cloned into the BamHI/PstI sites of pTRB30	83,84	This study
pOocM	Km^r; *oocM* gene was cloned into the SacI/PstI sites of pTRB30	85,86	This study
pOocO	Km^r; *oocO* gene was cloned into the BamHI/PstI sites of pTRB30	87,88	This study
pOocP	Km^r; *oocP* gene was cloned into the BamHI/SalI sites of pTRB30	89,90	This study

pOocQ	Km^r; *oocQ* gene was cloned into the BamHI/KpnI sites of pTRB30	91,92	This study
pOocU	Km^r; *oocU* gene was cloned into the BamHI/PstI sites of pTRB30	93,94	This study
pOocV	Km^r; *oocV* gene was cloned into the SphI/PstI sites of pTRB30	95,96	This study
pAT1	Km^r; *oocV* gene with catalytic serine of AT1 domain mutated to alanine was cloned into the SphI/PstI sites of pTRB30	95/97 and 98/96	This study
pAT2	Km^r; *oocV* gene with catalytic serine of AT2 domain mutated to alanine was cloned into the SphI/PstI sites of pTRB30	95/99 and 100/96	This study
pOocW	Km^r; *oocW* gene was cloned into the EcoRI site of pTRB30	101,102	This study
pAT3	Km^r; *oocW* gene with catalytic serine of AT3 domain mutated to alanine was cloned into the EcoRI site of pTRB30	101/103 and 104/102	This study
pHfq	Sm^r; *hfq* gene was cloned into the BamHI/SalI sites of pTA100	105,106	This study
pRpoS	Sm^r; *rpoS* gene was cloned into the SphI/HindIII sites of pTA100	107,108	This study
pSplI	Km^r; *splI* gene was cloned into the BamHI/PstI sites of pTRB30	109,110	This study
pMAMV165	Tc^r; *oocG* promoter region was cloned into the KpnI/PstI sites of pMP220	111,112	This study
pMAMV166	Tc^r; *oocJ* promoter region was cloned into the KpnI/PstI sites of pMP220	113,114	This study

[a] Ap, ampicillin; Km, kanamycin; Sm, streptomycin; Sp, spectinomycin; Tc, tetracycline.

Supplementary Table S4. Oligonucleotides used in this study.

Number	Name	Sequence (5´- 3')	Description	Source
1	LacZ-EcoRI-F	TAATGAATTCTCCGTGGGGAAGACGTAC	Forward primer to clone a region of *lacZ* of A153 into pUC18	This study
2	LacZ-HindIII-R	TAATAAGCTTGAAGAGGCCAGGGACAAC	Reverse primer to clone a region of *lacZ* of A153 into pUC18	This study
3	OocA-EcoRI-F	AAAAGAATTCTTTACCCGAAACTGCACG	Forward primer to clone upstream flanking region of *oocA* for in-frame deletion	This study
4	OocA-BamHI-R	AAAAGGATCCTCTCATGGAGGAGTGACGCAA	Reverse primer to clone upstream flanking region of *oocA* for in-frame deletion	This study
5	OocA-BamHI-F	AAAAGGATCCGCAAAGTCTGGCCACGGATT	Forward primer to clone downstream flanking region of *oocA* for in-frame deletion	This study
6	OocA-PstI-R	AAAACTGCAGAACAGCAATGTATTGGTCG	Reverse primer to clone downstream flanking region of *oocA* for in-frame deletion	This study
7	OocB-EcoRI-F	AAAAGAATTCCGGCCAAGGCTTGTTCGATTC	Forward primer to clone upstream flanking region of *oocB* for in-frame deletion	This study
8	OocB-BamHI-R	AAAAGGATCCGGACACTCAACAGCAATGCAATGT	Reverse primer to clone upstream flanking region of *oocB* for in-frame deletion	This study
9	OocB-BamHI-F	TAATGGATCCGCAGCGCCTCGCTGGTTAT	Forward primer to clone downstream flanking region of *oocB* for in-frame deletion	This study
10	OocB-HindIII-R	TAATAAGCTTCCAGATACGCATGCAAGATCGC	Reverse primer to clone downstream flanking region of *oocB* for in-frame deletion	This study
11	OocE-SalI-F	CACATTCGTCGACAAAGCCGCTGA	Forward primer to clone upstream flanking region of *oocE* for in-frame deletion	This study
12	OocE-HindIII-R	GCGTAAGCTTCGGCACCAAAAAC	Reverse primer to clone upstream flanking region of *oocE* for in-frame deletion	This study
13	OocE-HindIII-F	GACATTGATAAAGCTTGTGCCGCAA	Forward primer to clone downstream flanking region of *oocE* for in-frame deletion	This study
14	OocE-ApaI-R	TTTGCCGGGCCCTTTTGATCATAATCG	Reverse primer to clone downstream flanking region of *oocE* for in-frame deletion	This study
15	OocK-EcoRI-F	AAAAGAATTCATCTGAGGGCGCTGGAAGCAC	Forward primer to clone upstream flanking region of *oocK* for in frame deletion	This study
16	OocK-BamHI-R	GGGATAATGAGGATCCTCCGGCA	Reverse primer to clone upstream flanking region of *oocK* for in-frame deletion	This study
17	OocK-BamHI-F	AAAAGGATCCATTCCTGGACAGCAAATATCTGG	Forward primer to clone downstream flanking region of *oocK* for in-frame deletion	This study
18	OocK-PstI-R	AAAACTGCAGAGGAGGCGGAAAGTGACACG	Reverse primer to clone downstream flanking region of *oocK* for in-frame deletion	This study
19	OocM-EcoRI-F	AAAAGAATTCGCGGATTATCTCTCTTCCTTGTGC	Forward primer to clone upstream flanking region of *oocM* for in-frame deletion	Matilla *et al*., (2012)
20	OocM-BamHI-R	GATCCGAGGGATCCTTGCGGACATCAG	Reverse primer to clone upstream flanking region of *oocM* for in-frame deletion	This study
21	OocM-BamHI-F	ACTTCGGCATGGATCCCGACGC	Forward primer to clone downstream flanking region of *oocM* for in-frame deletion	This study
22	OocM-PstI-R	TTTTCTGCAGCATACTCATTGGATTCGCAGCCG	Reverse primer to clone downstream flanking region of *oocM* for in-frame deletion	Matilla *et al*., (2012)
23	OocO-EcoRI-F	AAAAGAATTCCATGAGCATTGGCGGC	Forward primer to clone upstream flanking region of *oocO* for in-frame deletion	This study
24	OocO-BamHI-R	AAAAGGATCCCCGACAAGGCTTCGTCTAATTC	Reverse primer to clone upstream flanking region of *oocO* for in-frame deletion	This study
25	OocO-BamHI-F	AAAAGGATCCACTTCAATCGTCTTGAAATGACCT	Forward primer to clone downstream flanking region of *oocO* for in-frame deletion	This study
26	OocO-PstI-R	AAAACTGCAGGTGCGAAGATTGCCGA	Reverse primer to clone downstream flanking region of *oocO* for in-frame deletion	This study

27	OocP-EcoRI-F	AAAAGAATTCAGGATGGCATTTACGACAGC	Forward primer to clone upstream flanking region of *oocP* for in-frame deletion	This study
28	OocP-BamHI-R	AAAAGGATCCAAGTGTGGCATCAGGTCGAAG	Reverse primer to clone upstream flanking region of *oocP* for in-frame deletion	This study
29	OocP-BamHI-F	TAATGGATCCTCTGGATACCATGACCAAGGG	Forward primer to clone downstream flanking region of *oocP* for in-frame deletion	This study
30	OocP-HindIII-R	TAATAAGCTTCGTCACTTGTTTCTCCA	Reverse primer to clone downstream flanking region of *oocP* for in-frame deletion	This study
31	OocQ-EcoRI-F	AAAAGAATTCGGCAATCTTCGCACCTG	Forward primer to clone upstream flanking region of *oocQ* for in-frame deletion	This study
32	OocQ-HindIII-R	TAATAAGCTTGAACTCACCGGAATTTATCAGGTAC	Reverse primer to clone upstream flanking region of *oocQ* for in-frame deletion	This study
33	OocQ-HindIII-F	TAATAAGCTTGTTCGTGACAGCCAAACGG	Forward primer to clone downstream flanking region of *oocQ* for in-frame deletion	This study
34	OocQ-PstI-R	AAAACTGCAGCCTTGGCGAGAAACGG	Reverse primer to clone downstream flanking region of *oocQ* for in-frame deletion	This study
35	OocT-EcoRI-F	CTCAGAGACCGGTATGAATTCGAGC	Forward primer to clone upstream flanking region of *oocT* for in-frame deletion	This study
36	OocT-HindIII-R	TTTTAAGCTTGGGCTATCCGCATACGCATTG	Reverse primer to clone upstream flanking region of *oocT* for in-frame deletion	This study
37	OocT-HindIII-F	TTTTAAGCTTGGCGACACGGGATTTTCCAG	Forward primer to clone downstream flanking region of *oocT* for in-frame deletion	This study
38	OocT-PstI-R	AGAACATCTGCAGTGCCTTGATCGG	Reverse primer to clone downstream flanking region of *oocT* for in-frame deletion	This study
39	OocU-SalI-F	CGCTGGTCGACAAAAACGCATT	Forward primer to clone upstream flanking region of *oocU* for in-frame deletion	This study
40	OocU-HindIII-R	CGACAAGCTTGCGGGATGCAATT	Reverse primer to clone upstream flanking region of *oocU* for in-frame deletion	This study
41	OocU-HindIII-F	TTTTAAGCTTTTGATGCAGGAAGCCGCCTC	Forward primer to clone downstream flanking region of *oocU* for in-frame deletion	This study
42	OocU-ApaI-R	ATAAGGGCCCTGGAAAGTAAACTGCG	Reverse primer to clone downstream flanking region of *oocU* for in-frame deletion	This study
43	OocV-AT1-EcoRI-F	ATATGAATTCGGCTCGGCAAGGCTA	Forward primer to clone upstream flanking region of AT1 domain of *oocV* for in-frame deletion	This study
44	OocV-AT1-BamHI-R	AAAAGGATCCAAATTCTGCGCCATCCGCTTAA	Reverse primer to clone upstream flanking region of AT1 domain of *oocV* for in-frame deletion	This study
45	OocV-AT1-BamHI-F	AAAAGGATCCATGTTCGCGGGCCAGGGG	Forward primer to clone downstream flanking region of AT1 domain of *oocV* for in-frame deletion	This study
46	OocV-AT1-PstI-R	AAAACTGCAGCTTGTTGCATCTCGCCG	Reverse primer to clone downstream flanking region of AT1 domain of *oocV* for in-frame deletion	This study
47	OocV-AT2-EcoRI-F	TAATGGTACCGCGGATGGCGCAGAATTTATG	Forward primer to clone upstream flanking region of AT2 domain of *oocV* for in-frame deletion	This study
48	OocV-AT2-HindIII-R	TAATAAGCTTGAAAACGAACTTGTGCGGCG	Reverse primer to clone upstream flanking region of AT2 domain of *oocV* for in-frame deletion	This study
49	OocV-AT2-HindIII-F	TAATAAGCTTTATTCGCCGGATTTTACCTGTCG	Forward primer to clone downstream flanking region of AT2 domain of *oocV* for in-frame deletion	This study
50	OocV-AT2-PstI-R	AAAACTGCAGCTGGAGTGAAAAGCACCGCTG	Reverse primer to clone downstream flanking region of AT2 domain of *oocV* for in-frame deletion	This study
51	OocV-AT2-BamHI-F	TAATGGATCCTATTCGCCGGATTTTACCTGTCG	Forward primer to clone downstream region of AT2 for in-frame deletion of *oocV*	This study

52	OocW-EcoRI-km3-F	AAAAGAATTCGATGCTCCGGCTGGACGACG	Forward primer to mutagenesis of *oocW*	This study
53	OocW-PstI-km3-R	AAAACTGCAGCGTAGCGCCGTGCTTAAC	Reverse primer to mutagenesis of *oocW*	This study
54	oocVW-BamHI-R	TAATGGATCCTGCGATACTGCGATCCCTGAC	Reverse primer to clone upstream flanking region of *oocV* for in-frame deletion of *oocW-oocW*	This study
55	oocVW-BamHI-F	TAATGGATCCCTTTGTTGCTGTTCCAACATCGC	Forward primer to clone downstream flanking region of *oocW* for in-frame deletion of *oocW-oocW*	This study
56	oocVW-HindIII-R	TAATAAGCTTGGCAGAGTATCGGCAGTTGC	Reverse primer to clone downstream flanking region of *oocW* for in-frame deletion of *oocW-oocW*	This study
57	ORF10ACPL-EcoRI-F	AAAAGAATTCAGCATCCTGAGCAGGG	Forward primer to clone upstream region of ACP_L encoding domain of *oocJ* to generate a point mutation in the G*X*DS motif of the ACP_L domain of OocJ	This study
58	ORF10ACPL-R	AATGGCGGCGAAGGCGCCGACGCCATACTCGG	Reverse primer to clone upstream region of ACP_L domain encoding domain of *oocJ* to generate a point mutation in the G*X*DS motif of the ACP_L domain of OocJ. Overlapping PCR primer	This study
59	ORF10ACPL-F	CCGAGTATGGCGTCGGCGCCTTCGCCGCCATT	Forward primer to clone downstream region of ACP_L domain encoding domain of *oocJ* to generate a point mutation in the G*X*DS motif of the ACP_L domain of OocJ. Overlapping PCR primer	This study
60	ORF10ACPL-HindIII-R	GCTTCTCAAGCTTTCGCAGG	Reverse primer to clone downstream region of ACP_L domain encoding of *oocJ* to generate a point mutation in the G*X*DS motif of the ACP_L domain of OocJ	This study
61	OocS-TE-EcoRI-F	AAAAGAATTCCAGGGTAAACGCTACTGGG	Forward primer to clone upstream region of TE encoding domain of *oocS* to generate a point mutation in the G*X*S*X*G motif of the TE domain of OocS	This study
62	OocS-TE-R	TAATCCCGCCCATTGCATAGCCTGCCAG	Reverse primer to clone upstream region of TE encoding domain of *oocS* to generate a point mutation in the G*X*S*X*G motif of the TE domain of OocS. Overlapping PCR primer	This study
63	OocS-TE-F	CTGGCAGGCTATGCAATGGGCGGGATTA	Forward primer to clone downstream region of TE encoding domain of *oocS* to generate a point mutation in the G*X*S*X*G motif of the TE domain of OocS. Overlapping PCR primers	This study
64	OocS-TE-PstI-R	AAAACTGCAGGTTCTTCCGCATCGGC	Reverse primer to clone downstream region of TE encoding domain of *oocS* to generate a point mutation in the G*X*S*X*G motif of the TE domain of OocS	This study
65	OocS-Cond-EcoRI-F	TAATGAATTCGATGCCGAGCTGCTGAC	Forward primer to clone upstream region of C encoding domain of *oocS* to generate a point mutation in the HH*XXX*DG motif of the C domain of OocS	This study
66	OocS-Cond-R	AGCATGAACAAGGTAAAACCTGCGGCATGGAAGGCAGC	Reverse primer to clone upstream region of C encoding domain of *oocS* to generate a point mutation in the HH*XXX*DG motif of the C domain of OocS. Overlapping PCR primer	This study
67	OocS-Cond-F	GCTGCCTTCCATGCCGCAGGTTTTACCTTGTTCATGCT	Forward primer to clone downstream region of C encoding domain of *oocS* to generate a point mutation in the HH*XXX*DG motif of the C domain of OocS. Overlapping PCR primer	This study
68	OocS-Cond-HindIII-R	TAATAAGCTTCCAGCGACATCGGCAGC	Reverse primer to clone downstream region of C encoding domain of *oocS* to generate a point mutation in the HHNNNDG motif of the C domain of OocS	This study
69	Hfq-EcoRI-F	GTTGCGGCAATGAGAATTCATCC	Forward primer to clone upstream flanking region of *hfq* for gene deletion A153 and 4Rx5	This study
70	Hfq-BamHI-R	AAAAGGATCCGAACGGATCTTGCAAAGATTGCC	Reverse primer to clone upstream flanking region of *hfq* for gene deletion A153 and 4Rx5	This study

71	Hfq-BamHI-F	AAAAGGATCCCAGGAAAGCGATGACGCTGAAT	Forward primer to clone downstream flanking region of *hfq* for gene deletion in A153 and 4Rx5	This study
72	Hfq-SphI-R	AAAAGCATGCCGGCGTTGGTATAACCC	Reverse primer to clone downstream flanking region of *hfq* for gene deletion A153 and 4Rx5	This study
73	RpoS-EcoRI-F	TAATGAATTCTTCGCTACCGCCGATGGTC	Forward primer to clone a region of *rpoS* of A153 into pUC18	This study
74	RpoS-HindIII-R	TAATAAGCTTCGCCACACAGATGACGCTGC	Reverse primer to clone a region of *rpoS* of A153 into pUC18	This study
75	LuxI-4Rx5-EcoRI-F	TAATGAATTCTCGCTTGGGAACACAAGAACCT	Forward primer to clone upstream flanking region of *splI* for in-frame deletion in 4Rx5	This study
76	LuxI-4Rx5-BamHI-R	TAATGGATCCCGTTCGAGTGCGTCACTTTCAG	Reverse primer to clone upstream flanking region of *splI* for in-frame deletion in 4Rx5	This study
77	LuxI-4Rx5-BamHI-F	TAATGGATCCGGCTTGGTTGTCCGAGATCGTC	Forward primer to clone downstream flanking region of *splI* for in-frame deletion in 4Rx5	This study
78	LuxI-4Rx5-HindIII-R	TAATAAGCTTCGGGACCATATCGACAGGAAG	Reverse primer to clone downstream flanking region of *splI* for in-frame deletion in 4Rx5	This study
79	OocC-BamHI-F	TAATGGATCCTCGACGAATGTCATTGATTTTCAG	Forward primer to clone *oocC* into pTA100	This study
80	OocC-HindIII-R	TAATAAGCTTGCATGCGTTAATTGCCAAATAG	Reverse primer to clone *oocC* into pTA100	This study
81	E-BamHI-F	TAATGGATCCATTTCCGTTGGAATTGAAGCG	Forward primer to clone *oocE* into pTRB30	This study
82	E-SalI-R	TAATGTCGACGCGTTTGATATGACGACATGG	Reverse primer to clone *oocE* into pTRB30	This study
83	K-BamHI-F	AAAAGGATCCGATGTACAAGGAAAACGAGTATGC	Forward primer to clone *oocK* into pTRB30	This study
84	K-PstI-R	AAAACTGCAGGCTGACTCGTGTAAGTCATGG	Reverse primer to clone *oocK* into pTRB30	This study
85	M-SacI-F	AAAAGAGCTCAATACGCTGAATCAAAAGCTGG	Forward primer to clone *oocM* into pTRB30	This study
86	M-PstI-R	AAAACTGCAGGATAATTGTGCTGCTTGCTTAC	Reverse primer to clone *oocM* into pTRB30	This study
87	O-BamHI-F	TAATGGATCCAATGAATTAGACGAAGCCTTGTC	Forward primer to clone *oocO* into pTRB30	This study
88	O-PstI-R	TAATCTGCAGAGTTTCCTCTGAGACTCATGTTC	Reverse primer to clone *oocO* into pTRB30	This study
89	P-BamHI-F	TAATGGATCCGAAAAGTATGACATTCAAAGTGAAACG	Forward primer to clone *oocP* into pTRB30	This study
90	P-SalI-R	TAATGGTACCGGTACTCGCTCATTGTTTATCCC	Reverse primer to clone *oocP* into pTRB30	This study
91	Q-BamHI-F	TAATGGATCCAGCGAGTACCTGATAAATTCCG	Forward primer to clone *oocQ* into pTRB30	This study
92	Q-KpnI-R	TAATGGTACCCAAGCGAATCGGTTACTAACG	Reverse primer to clone *oocQ* into pTRB30	This study
93	U-BamHI-F	AAAAGGATCCACCTATGTGAAAACGCTGGC	Forward primer to clone *oocU* into pTRB30	This study
94	U-PstI-R	AAAACTGCAGGCGCCATCCGCTTAAGAATAG	Reverse primer to clone *oocU* into pTRB30	This study
95	V-SphI-F	AAAAGCATGCAAAACAGTCTATACCTTTCCTGGTC	Forward primer to clone *oocV* into pTRB30	This study
96	V-PstI-R	AAAACTGCAGTATTAGCCACCACCCCTGTTAC	Reverse primer to clone *oocV* into pTRB30	This study
97	V-AT1-R	GTTATACTCTCCCAGAGCGTGCCCGGCC	Reverse primer to mutate catalytic serine of AT1 domain of *oocV*. Overlapping PCR primer	This study
98	V-AT1-F	GGCCGGGCACGCTCTGGGAGAGTATAAC	Forward primer to mutate catalytic serine of AT1 domain of *oocV*. Overlapping PCR [illegible]	This study

99	V-AT2-R	GTATTCCCCCAAAGCGTGGCCGACCA	Reverse primer to mutate catalytic erine of AT2 domain of *oocV*. Overlapping PCR primer	This study
100	V-AT2-F	TGGTCGGCCACGCTTTGGGGGAATAC	Forward primer to mutate catalytic serine of AT2 domain of *oocV*. Overlapping PCR primer	This study
101	W-RBS-EcoRI-F	TAATGAATTCAATGAGAAGGAGTGTACGACTATGCCAAT TCGAGTTTATATGTTCCCGGGACAGGGCTC	Forward primer to clone *oocW* into pTRB30; includes ribosome binding site.	This study
102	W-EcoRI-R	TAATGAATTCCCGCCATGTCGTGTCGTCAAAC	Reverse primer to clone *oocW* into pTRB30	This study
103	W-AT3-R	TGAACTCCCCCAGGGCATGCCCTAAA	Reverse primer to mutate catalytic serine of AT3 domain of *oocW*; overlapping PCR primer	This study
104	W-AT3-F	TTTAGGGCATGCCCTGGGGGAGTTCA	Forward primer to mutate catalytic serine of AT3 domain of *oocW*; overlapping PCR	This study
105	A4Hfq-BamHI-F	TAATGGATCCGCTAAGGGGCAATCTTTGCA	Forward primer to clone *hfq* into pTA100	This study
106	A4Hfq-HindIII-F	TAATAAGCTTCTCGCAACGCGCTTTATTC	Reverse primer to clone *hfq* into pTA100	This study
107	RpoS-SphI-F	AAAAGCATGCAACCAAAATACGCTGAAAGTTAACG	Forward primer to clone *rpoS* into pTA100	This study
108	RpoS-HindIII-R	AAAAAAGCTTGATTCAACTGCCGACGTTATTC	Reverse primer to clone *rpoS* into pTA100	This study
109	SplI-4Rx5-BamHI-F	TAATGGATCCCTTGAACTATTTGATGTTAGTTACGAAG	Forward primer to clone *splI* into pTRB30	This study
110	SplI-4Rx5-PstI-R	TAATCTGCAGGGCGTTGAGTTGGAACTTAT	Reverse primer to clone *splI* into pTRB30	This study
111	Pmt-OocG-KpnI-F	TAATGGTACCCAGGCGCTAACTACAGGTGAC	Forward primer to clone promoter region of *oocG* into pMP220	This study
112	Pmt-OocG-PstI-R	TAATCTGCAGCATACACTGCTCCTTACTTGCATGG	Reverse primer to clone promoter region of *oocG* into pMP220	This study
113	Pmt-OocJ-KpnI-F	TAATGGTACCTCTTTACGCTGTCAGAGTCGC	Forward primer to clone promoter region of *oocJ* into pMP220	This study
114	Pmt-OocJ-PstI-R	TAATCTGCAGCATCACGACCCCTTCACCAA	Reverse primer to clone promoter region of *oocJ* into pMP220	This study
115	oocE-qPCR-F	GAATATCGGCATTCGCCAAC	Forward primer for qRT-PCR. Valid for A153 and 4Rx5	This study
116	oocE-qPCR-R	CCGCCGACATTGATTATGTG	Reverse primer for qRT-PCR. Valid for A153 and 4Rx5	This study
117	oocJ-qPCR-F	ATGCTTAAGTCTTCCGGGC	Forward primer for qRT-PCR. Valid for A153 and 4Rx5	This study
118	oocJ-qPCR-R	CATCATCTGAGCGACTTGC	Reverse primer for qRT-PCR. Valid for A153 and 4Rx5	This study
119	rpoS-qPCR-F	GCAACGGAAGCGGAGTCATT	Forward primer for qRT-PCR. Valid for A153 and 4Rx5	This study
120	rpoS-qPCR-R	ATAGAGCTGCGTCGCATCCA	Reverse primer for qRT-PCR. Valid for A153 and 4Rx5	This study
121	16S-qPCR-F	ACGTTACTCGCAGAAGAAGC	Forward primer for qRT-PCR. Valid for A153 and 4Rx5	This study
122	16S-qPCR-R	CCACCGGTATTCCTCCAGAT	Reverse primer for qRT-PCR. Valid for A153 and 4Rx5	This study
123	OocAB-F	GTGAACAGCAGGCAAAAGACTATC	MSU97 PCR mapping forward primer product 1	Matilla *et al*., (2012)
124	OocAB-R	CCTGCGTATAACCTGCACAC	MSU97 PCR mapping reverse primer product 1	Matilla *et al*., (2012)
125	OocBC-F	GATTCCATCATTCCGCCATCG	MSU97 PCR mapping forward primer product 2	Matilla *et al*., (2012)

126	OocBC-R	AATAACTATCGCGGCGAGGC	MSU97 PCR mapping reverse primer product 2	Matilla *et al*., (2012)
127	OocCD-F	CCTGTAAGGCGAACAGGTCT	MSU97 PCR mapping forward primer product 3	Matilla *et al*., (2012)
128	OocCD-R	CAGCGGCTTAGCTGCTTATC	MSU97 PCR mapping reverse primer product 3	Matilla *et al*., (2012)
129	OocDE-F	CGATGTTGCCTCGCTCGATG	MSU97 PCR mapping forward primer product 4	Matilla *et al*., (2012)
130	OocDE-R	GGTGAGTATGACGCCTTGCT	MSU97 PCR mapping reverse primer product 4	Matilla *et al*., (2012)
131	OocEF-F	CCGAAGTCGATGCCTGACTC	MSU97 PCR mapping forward primer product 5	Matilla *et al*., (2012)
132	OocEF-R	GGCCACGCTGATACAGATGA	MSU97 PCR mapping reverse primer product 5	Matilla *et al*., (2012)
133	OocFG-F	TAACTGTAGTCCGCCAGGCT	MSU97 PCR mapping forward primer product 6	Matilla *et al*., (2012)
134	OocFG-R	AACCTTACCGCACCTGGATG	MSU97 PCR mapping reverse primer product 6	Matilla *et al*., (2012)
135	OocGH-F	GGTAATATCGCCGGCATCCA	MSU97 PCR mapping forward primer product 7	Matilla *et al*., (2012)
136	OocGH-R	CATTCAACCCCATACACCTGATT	MSU97 PCR mapping reverse primer product 7	Matilla *et al*., (2012)
137	OocHI-F	GAATGGTTGTTTCTCCATTGAACG	MSU97 PCR mapping forward primer product 8	Matilla *et al*., (2012)
138	OocHI-R	AACAGCAAGGTAACAGTTAAAGCA	MSU97 PCR mapping reverse primer product 8	Matilla *et al*., (2012)
140	OocIJ-F	GCATCCGACAGGCTTGCTTA	MSU97 PCR mapping forward primer product 9	Matilla *et al*., (2012)
141	OocIJ-F	CCGCAACGATAGGTGAGAAC	MSU97 PCR mapping reverse primer product 9	Matilla *et al*., (2012)
142	OocJK-F	CGCGATCGTGACCTATAAGC	MSU97 PCR mapping forward primer product 10	Matilla *et al*., (2012)
143	OocJK-R	GTCCGACTCAGCCTCATACA	MSU97 PCR mapping reverse primer product 10	Matilla *et al*., (2012)
144	OocKL-F	CGTGGTGGATTACCTCAAGGC	MSU97 PCR mapping forward primer product 11	Matilla *et al*., (2012)
145	OocKL-R	CACCGGCAATTTGAGTATCACG	MSU97 PCR mapping reverse primer product 11	Matilla *et al*., (2012)
146	OocLM-F	CTGCAGGACTACGGAATGGA	MSU97 PCR mapping forward primer product 12	Matilla *et al*., (2012)

147	OocLM-R	GCCGTTATTGGCGTAGATCG	MSU97 PCR mapping reverse primer product 12	Matilla *et al*., (2012)
148	OocMN-F	GTGGTCTGCGATCCGTTCAA	MSU97 PCR mapping forward primer product 13	Matilla *et al*., (2012)
149	OocMN-R	CTCGGCAATGCTCATGCACT	MSU97 PCR mapping reverse primer product 13	Matilla *et al*., (2012)
150	OocNO-F	CCGTGGATTGGTCTCTGTTG	MSU97 PCR mapping forward primer product 14	Matilla *et al*., (2012)
151	OocNO-R	ATACCACCGGAGTCAGCTTG	MSU97 PCR mapping reverse primer product 14	Matilla *et al*., (2012)
152	OocOP-F	GAGTCGATGGAGGTCAACCAAT	MSU97 PCR mapping forward primer product 15	Matilla *et al*., (2012)
153	OocOP-R	CCTGGAGGTCAGCCAACTTC	MSU97 PCR mapping reverse primer product 15	Matilla *et al*., (2012)
154	OocPQ-F	CATCAACACGACGCCACTGC	MSU97 PCR mapping forward primer product 16	Matilla *et al*., (2012)
155	OocPQ-R	AGATCTTGCCAGGATTCATCGG	MSU97 PCR mapping reverse primer product 16	Matilla *et al*., (2012)
156	OocQR-F	AATCGCTTTCCGAAGCGGAAC	MSU97 PCR mapping forward primer product 17	Matilla *et al*., (2012)
157	OocQR-R	GAGACGCTTGCTTATCCAGTTTG	MSU97 PCR mapping reverse primer product 17	Matilla *et al*., (2012)
158	OocRS-F	GGCTTCGCCTGTTTTGAACG	MSU97 PCR mapping forward primer product 18	Matilla *et al*., (2012)
159	OocRS-R	AGGCTATCAGTCCGGCTTCC	MSU97 PCR mapping reverse primer product 18	Matilla *et al*., (2012)
160	OocST-F	CCGTTCATCTACGACCGCAG	MSU97 PCR mapping forward primer product 19	Matilla *et al*., (2012)
161	OocST-R	TGGGTGATATTGATCACGCCG	MSU97 PCR mapping reverse primer product 19	Matilla *et al*., (2012)
162	OocTU-F	CCGCACTTACAATGACCGCT	MSU97 PCR mapping forward primer product 20	Matilla *et al*., (2012)
163	OocTU-R	GAAGAAGCTCATCATGTTCGCC	MSU97 PCR mapping reverse primer product 20	Matilla *et al*., (2012)
164	OocUV-F	GCGCCTTCAATCAATGGGTAA	MSU97 PCR mapping forward primer product 21	Matilla *et al*., (2012)
165	OocUV-R	GTTGCGTGTATTGCGTCTGG	MSU97 PCR mapping reverse primer product 21	Matilla *et al*., (2012)

166	OocVW-F	GGCAAGTTATCCGCGACAAG	MSU97 PCR mapping forward primer product 22	Matilla *et al*., (2012)
167	OocVW-R	AGAGCCGCTCGATGGAATAG	MSU97 PCR mapping reverse primer product 22	Matilla *et al*., (2012)

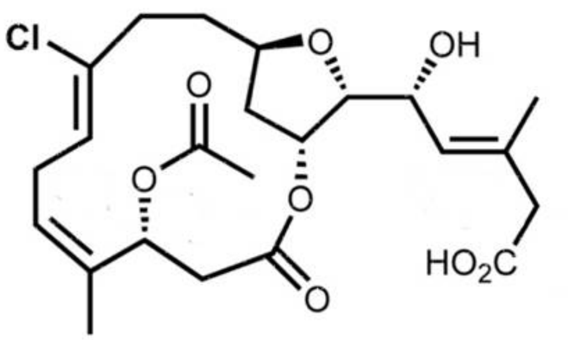

Supplementary Figure S1: Structure of oocydin A.

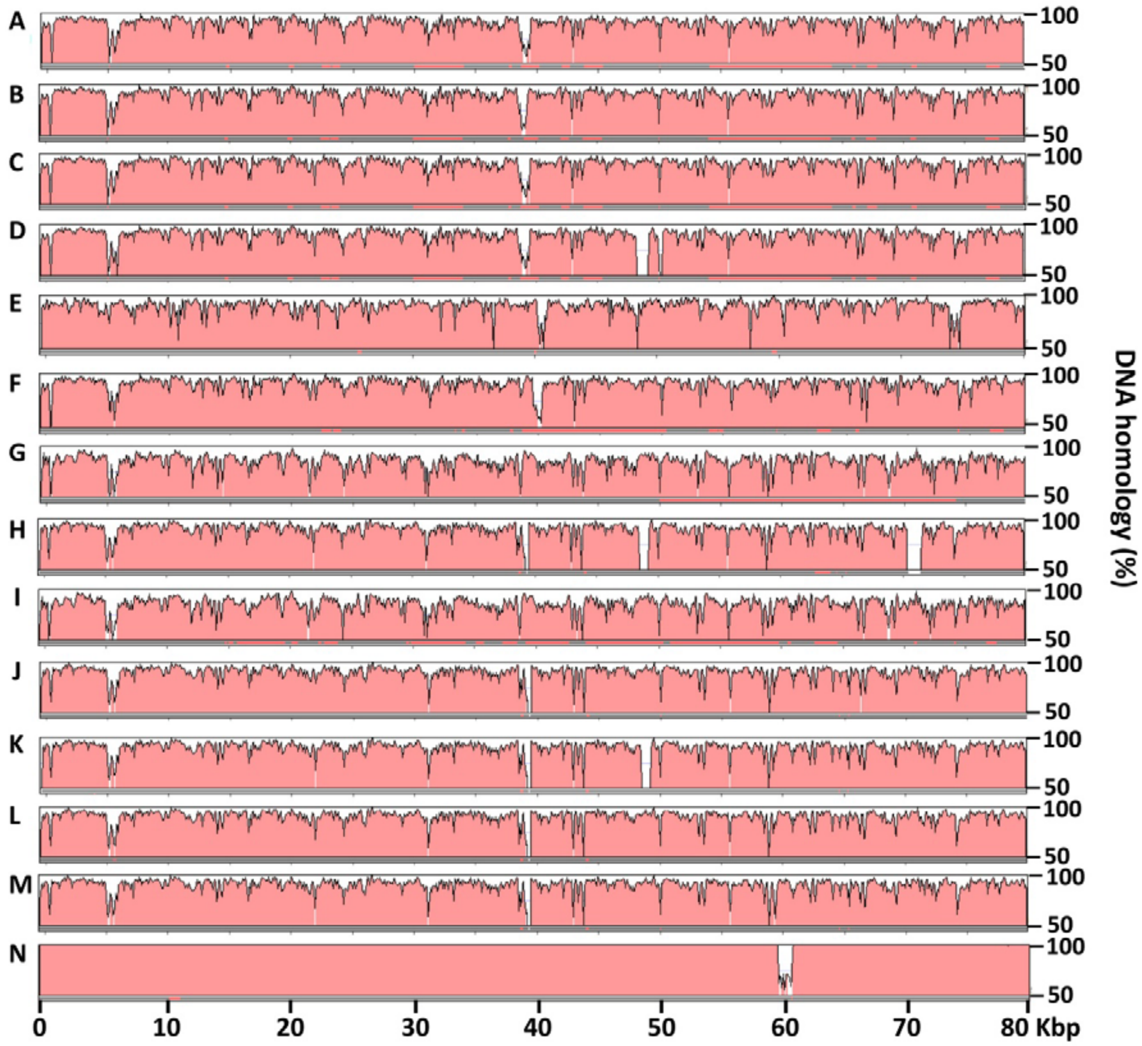

Supplementary Figure S2. The oocydin A gene cluster is widespread within the *Dickeya* genus. The alignments represent the percentage of DNA homology between the oocydin A (*ooc*) gene cluster of *Dickeya paradisiaca* Ech703 and the those of *Dickeya solani* strains MK10 (**A**), MK16 (**B**), IPO 2222 (**C**), GBBC 2040 (**D**), 3337 (**E**) and D_s0432-1 (**F**), *Dickeya chrysanthemi* NCPPB 402 (**G**), *Dickeya* sp. CSL RW240 (**H**), *Dickeya* sp. NCPPB 3274 (**I**), *Dickeya dianthicola* strains NCPPB 453 (**J**), GBBC 2039 (**K**), NCPPB 3534 (**L**), IPO 980 (**M**) and *Dickeya paradisiaca* NCPPB 2511 (**N**). Alignments were performed using wgVISTA (Frazer *et al*., 2004).

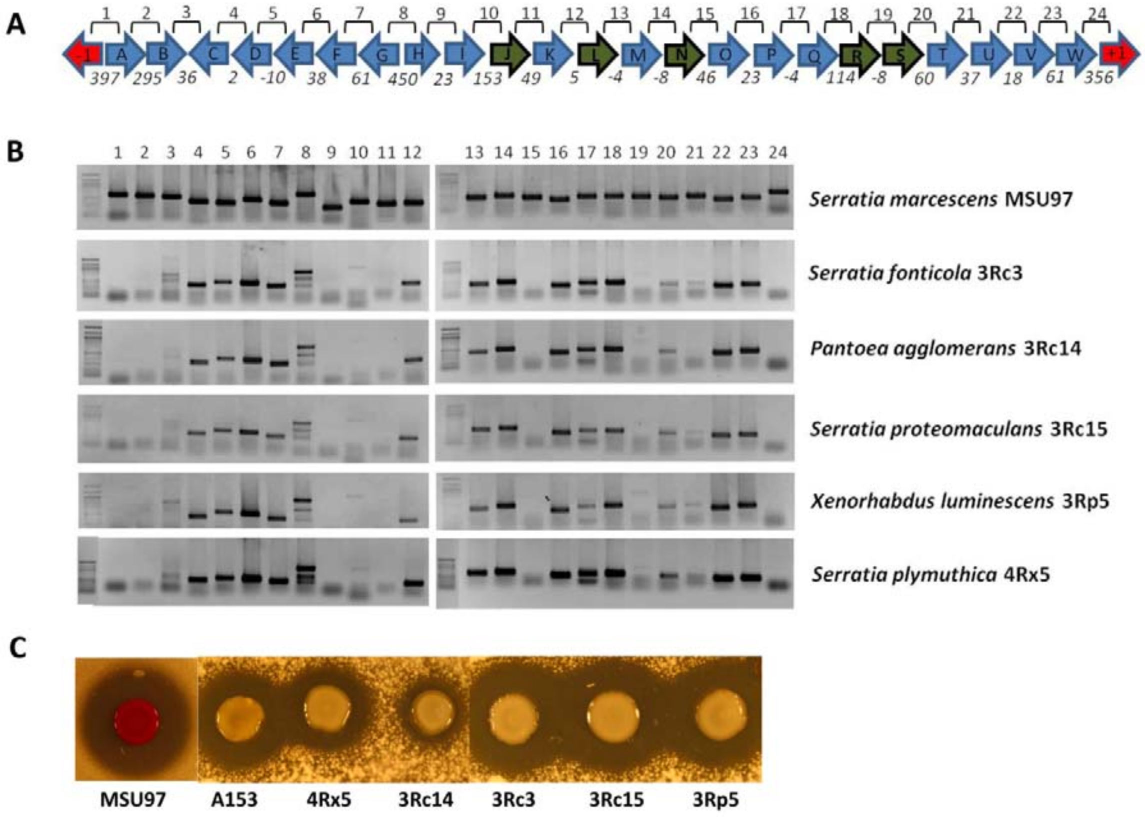

Supplementary Figure S3. The oocydin A gene cluster is present in several enterobacterial strains isolated from the rhizosphere of agronomically important crops. A, Representation of the *ooc* gene cluster in *Serratia marcescens* MSU97. Lines labeled 1-24 above the gene cluster represent the regions amplified in the PCR shown in **B**. Numbers below the arrows represent the intergenic distance between contiguous genes, with negative numbers indicate overlapping genes. Polyketide synthase encoding genes are shown in green. Genes flanking the *ooc* gene cluster are shown in red. **B**, Analysis by PCR using primers designed to span the intergenic region between two adjacent genes of the *ooc* gene cluster in MSU97. **C**, Antifungal activities against *Verticillium dahliae*. The bioassays were repeated at least three times, and representative results are shown. *Verticillium dahliae* pictures were taken after 96 h of incubation at 25 °C, respectively.

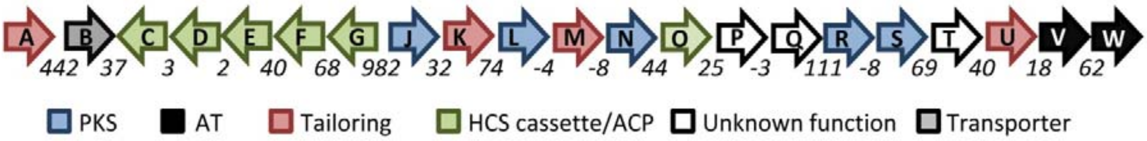

Supplementary Figure S4. Schematic representation of the oocydin A gene cluster in *Serratia plymuthica* 4Rx5. Numbers below the arrows represent the intergenic distance between contiguous genes, with negative numbers indicate overlapping genes.

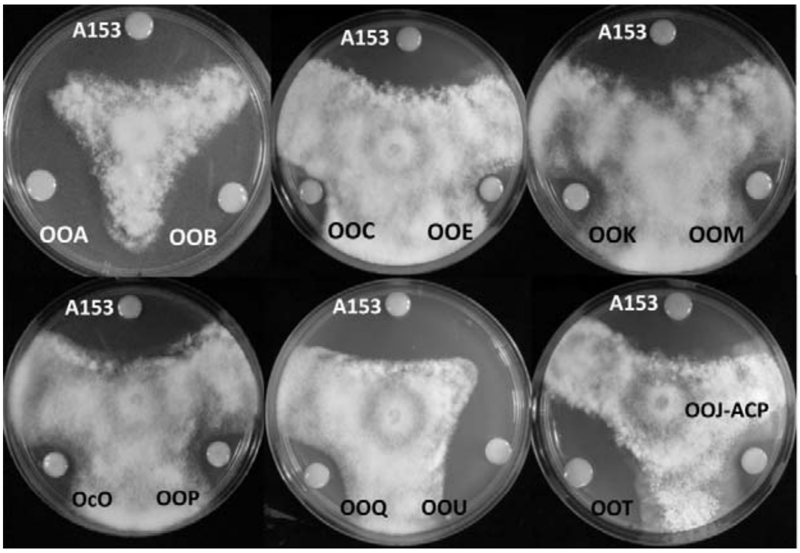

Supplementary Figure S5. Anti-oomycete activities of *Serratia plymuthica* A153 and derivative strains with mutations in the *ooc* gene cluster. Bioactivities of *S. plymuthica* A153 strains against *Pythium ultimum* are shown. The bioassays were repeated at least three times, and representative results are shown. Pictures were taken after 48 h of incubation at 25 °C. Complementation assays are shown in supplementary Fig. S6.

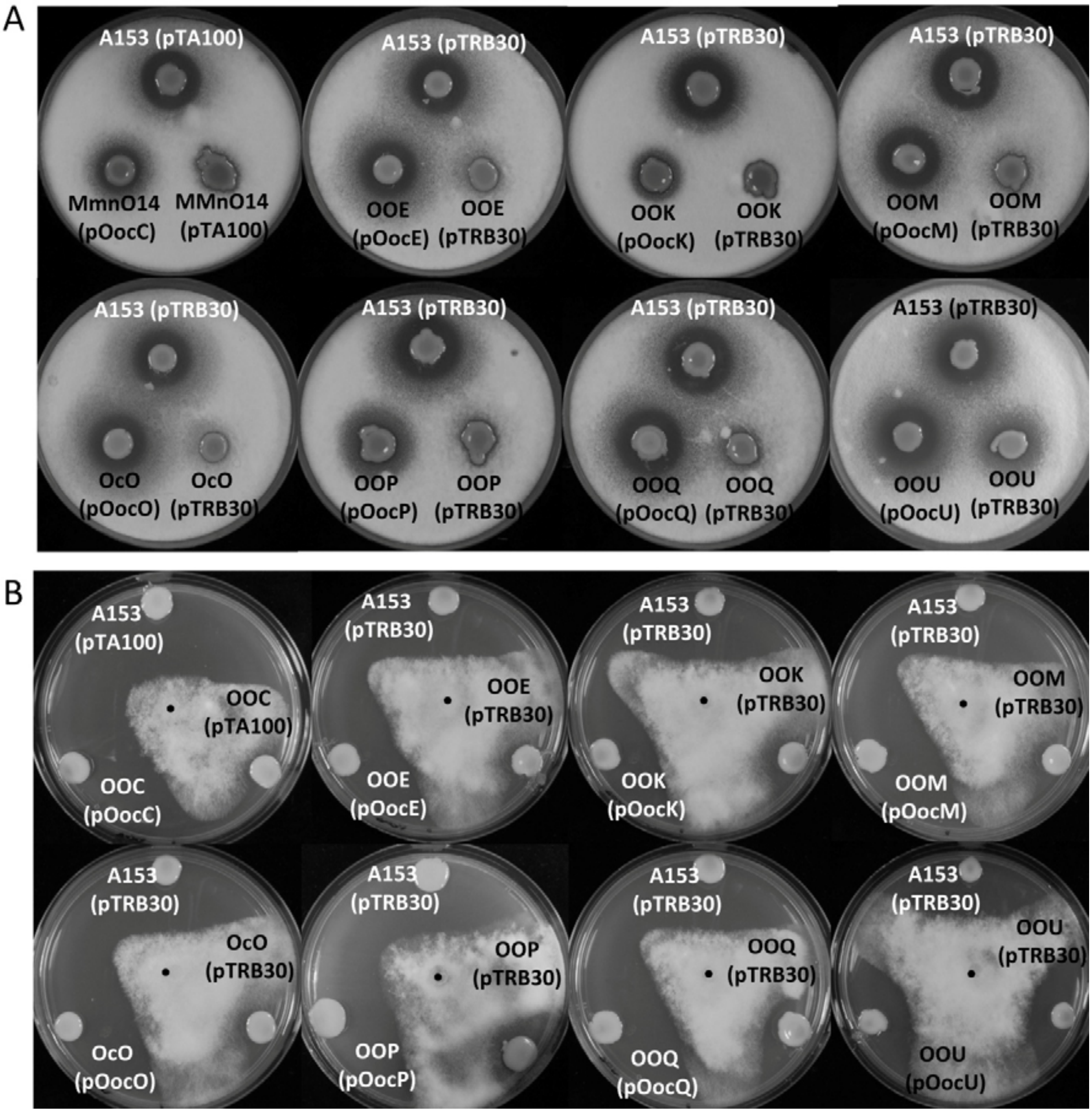

Supplementary Figure S6. Genetic complementation of *Serratia plymuthica* A153 mutants mapping to the *ooc* gene cluster. Bioactivities against *Verticillium dahliae* (**A**) and *Pythium ultimum* (**B**) are shown. *Pythium ultimum* inoculation points are indicated with a black dot. *Pythium ultimum* and *V. dahliae* pictures were taken after 48 and 96 h of incubation at 25 °C, respectively. Induction of the expression of the Ooc proteins was done by addition of 1 mM of IPTG.

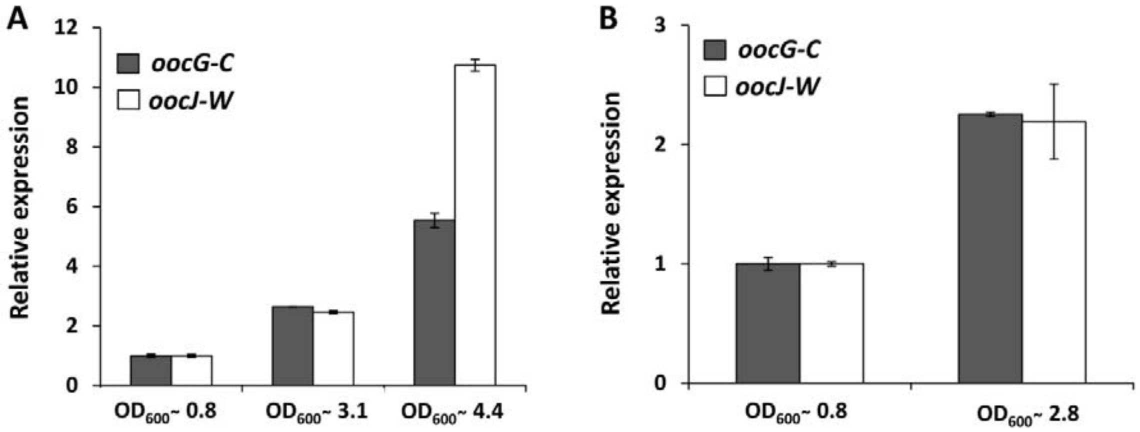

Supplementary Figure S7. Impact of growth phase on *ooc* transcript levels measured by qPCR. Measurement of *oocG-C* and *oocJ-W* transcript levels in *Serratia plymuthica* A153 (**A**) and *S. plymuthica* 4Rx5 (**B**) along the growth curve. The values showed the average expression relative to the expression at mid logarithmic phase of growth (OD_{600} ~ 0.8). Arrows in Fig. 4 and Fig. S10 indicate time points when the samples for qPCR were taken. Data are the mean and standard deviation of three biological replicates.

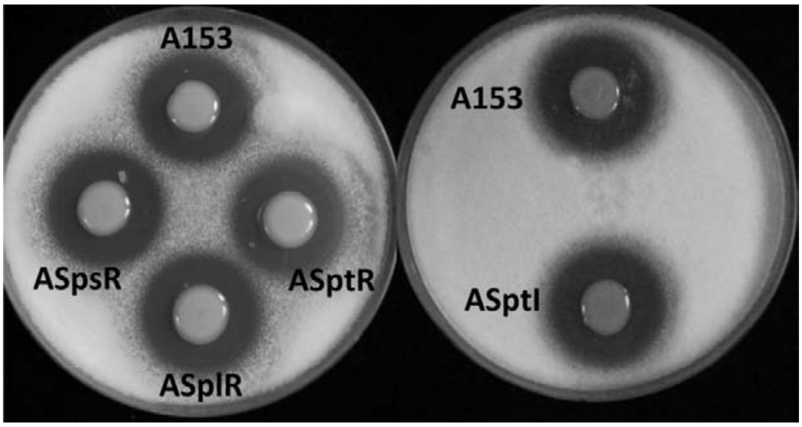

Supplementary Figure S8. An AHL-based quorum sensing system does not regulate the biosynthesis of oocydin A in *Serratia plymuthica* A153. Antifungal activities against *Verticillium dahliae* of *S. plymuthica* A153 strains are shown. The bioassays were repeated at least three times, and representative results are shown. Pictures were taken after 96 h of incubation at 25 °C.

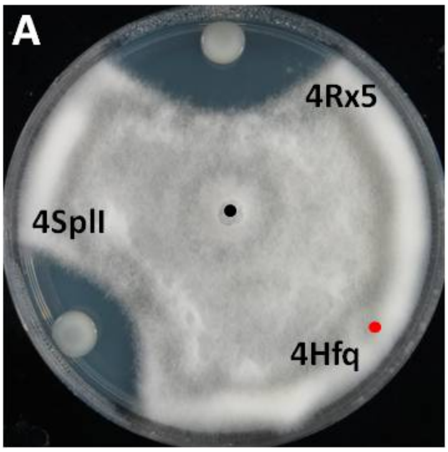

Supplementary Figure S9. Bioactivities of *Serratia plymuthica* 4Rx5 strains against *Pythium ultimum*. The inoculation points of *Pythium ultimum* and 4Hfq are indicated with a black or red dot, respectively. The bioassays were repeated three times, and representative results are shown. Pictures were taken after 48 and 72 h of incubation at 25 °C, respectively.

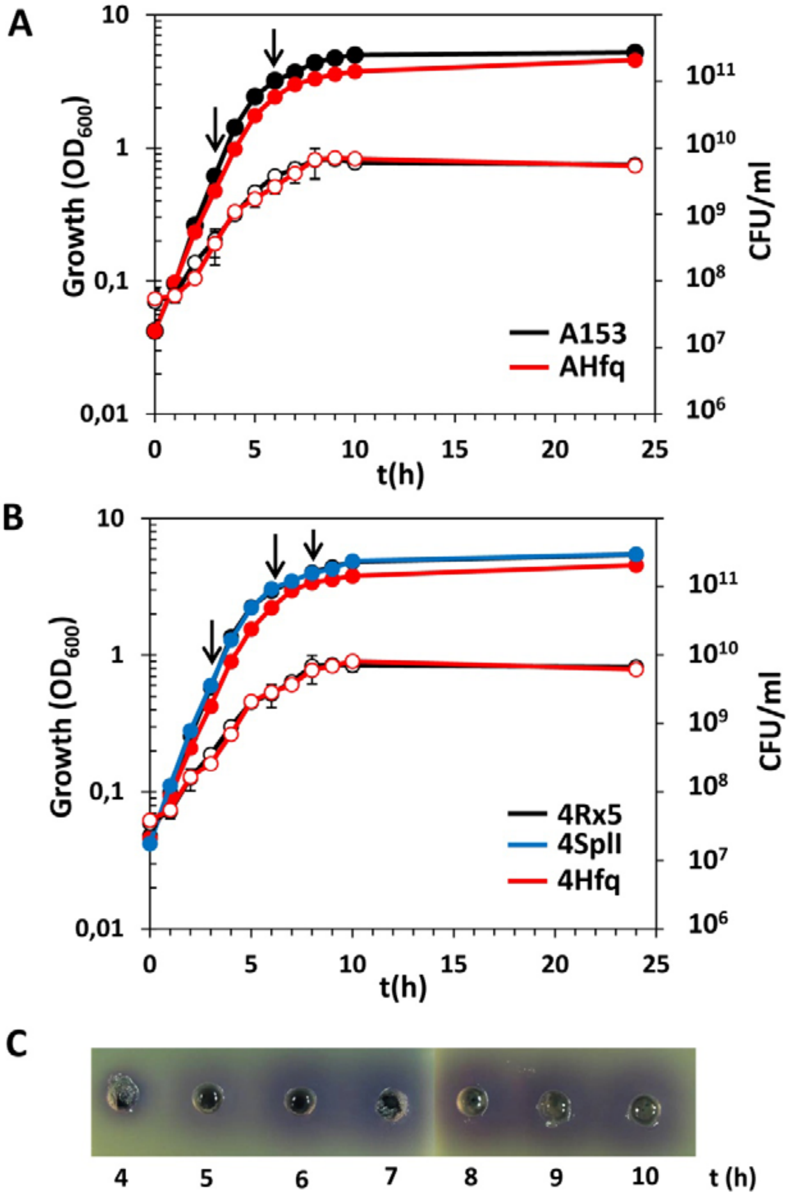

Supplementary Figure S10. Growth and quorum sensing signalling molecule production in *Serratia plymuthica* strains. Turbidity (filled symbols) and colony forming units per millilitre (CFU/ml; open symbols) of *Serratia plymuthica* A153 (**A**), *S. plymuthica* 4Rx5 (**B**), and their derivative strains, in LB medium at 25 ºC are shown. Data are the mean and standard deviation of three biological replicates. Arrows, time points where samples for quantitative RT-PCR were taken. **C**, Detection of AHLs in *S. plymuthica* 4Rx5 filter-sterilized supernatants throughout growth by biosensor strain CV026. Quorum sensing pictures were taken after 24 h of incubation at 25 °C.

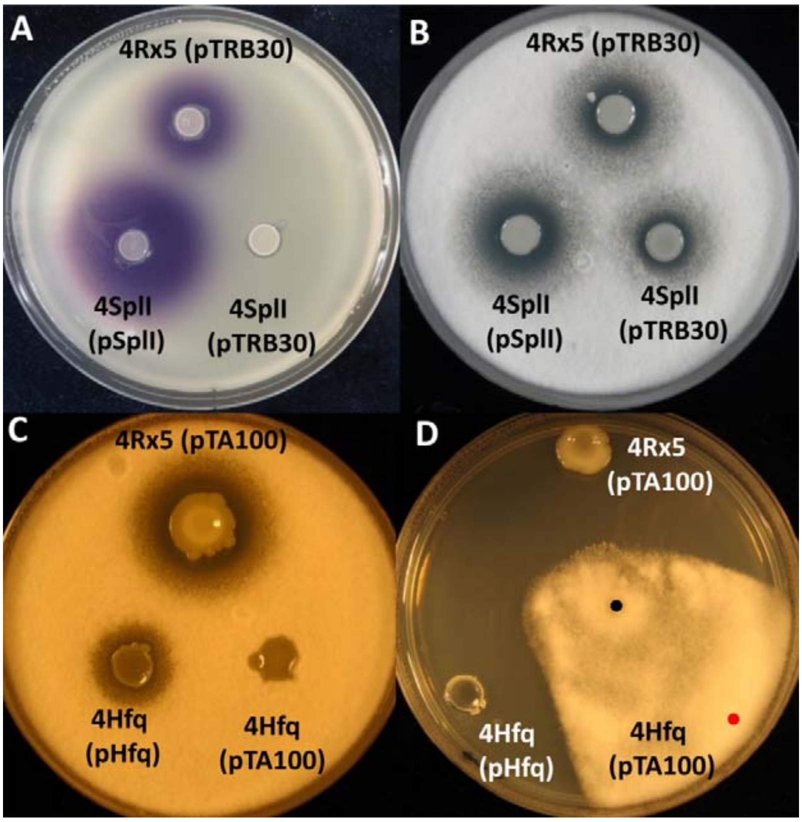

Supplementary Figure S11. Complementation assays of *Serratia plymuthica* 4Rx5 strains 4SplI and 4Hfq. Expression of *splI in trans* in the strain 4SplI restored AHLs production (**A**) and complemented the antifungal activity against *Verticillium dahliae* (**B**). Expression of *hfq in trans* complemented the antifungal (**C**) and anti-oomycete (**D**) activities in the strain 4Hfq. In D, the inoculation points of *Pythium ultimum* and 4Hfq are indicated with a black or red dot, respectively. Induction of the expression of the Hfq and SplI proteins was done by addition of 0.1 mM of IPTG. CV026, *Pythium ultimum* and *V. dahliae* pictures were taken after 48, 48 and 96 h of incubation at 25 °C, respectively.

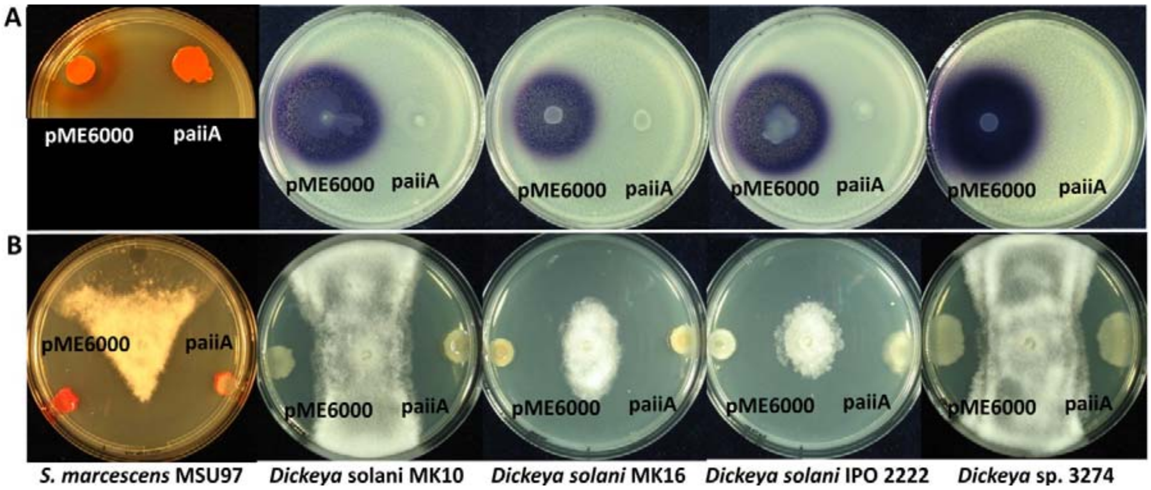

Supplementary Figure S12. An AHLs-based quorum sensing is not involved in the regulation of oocydin A in several enterobacteria strains. Effect of *aiiA* expression on the production of AHLs detected by using the biosensor strains SP19 (12) or CV026 (11) (**A**). Their bioactivities against the oomycete *Pythium ultimum* are also shown in **B**. The bioassays were repeated at least three times, and representative results are shown. CV026 and anti-oomycete pictures were taken after 48 h of incubation at 25 °C. SP19 pictures were taken after 48 h at 30 ºC.

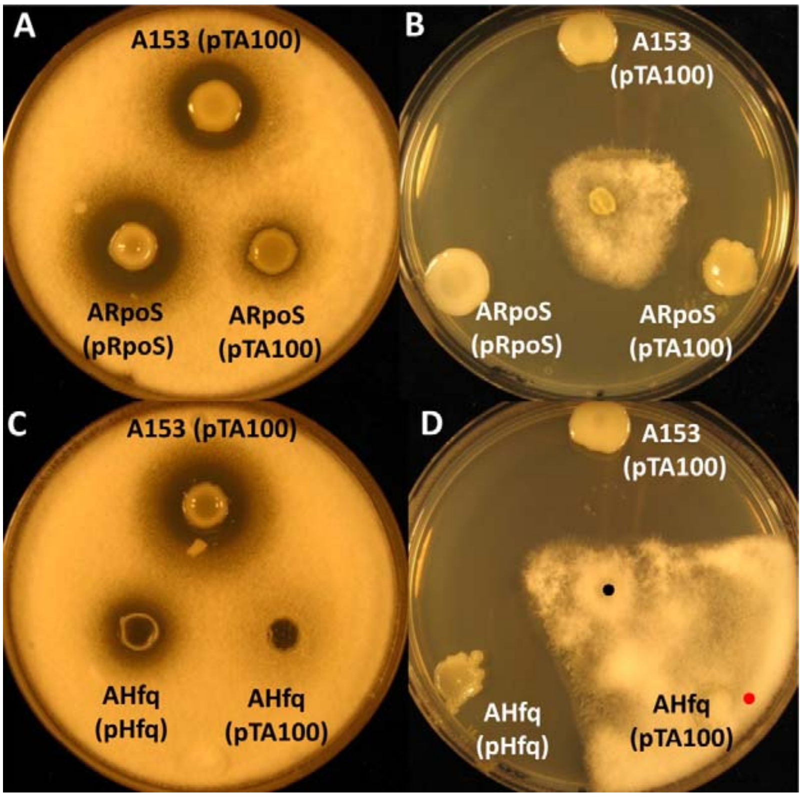

Supplementary Figure S13. Complementation assays of *Serratia plymuthica* A153 strains RpoS and AHfq. Expression of *rpoS in trans* in the strain ARpoS complemented the antifungal (**A**) and anti-oomycete (**B**) activities against *Verticillium dahliae* and *Pythium ultimum*, respectively. *In trans* expression of *hfq* complemented the antifungal (**C**) and anti-oomycete (**D**) activities in the strain AHfq. In D, the inoculation points of *Pythium ultimum* and AHfq are indicated with a black or red dot, respectively. Induction of the expression of the RpoS and SplI proteins was done by addition of 0.1 mM of IPTG. *Pythium ultimum* and *V. dahliae* pictures were taken after 48 and 96 h of incubation at 25 °C, respectively.

```
SM39            GCTCATAACGACACAATGCTGGTCCGGGAACAACAAGAAGTGAAGGCGGGTCAAAAAATA 60
Db11            GCTCATAACGACACAATGCTGGTCCGGGAACAACAAGAAGTGAAGGCGGGTCAAAAAATA 60
WW4             GCTCATAACGACACAATGCTGGTCCGGGAACAACAAGAAGTGAAGGCGGGTCAAAAAATA 60
ATCC_27592      GCTCACAACGACACAATGCTGGTCCGGGAACAACAAGAAGTGAAGGCGGGTCAAAAAATA 60
568             GCTCACAACGACACAATGCTGGTCCGGGAACAACAAGAAGTGAAGGCGGGTCAAAAAATA 60
A153            GCTCACAACGACACAATGCTGGTCCGGGAACAACAAGAAGTGAAGGCGGGTCAAAAAATA 60
4Rx5            GCTCACAACGACACAATGCTGGTCCGGGAACAACAAGAAGTGAAGGCGGGTCAAAAAATA 60
4Rx13           GCTCACAACGACACAATGCTGGTCCGGGAACAACAAGAAGTGAAGGCGGGTCAAAAAATA 60
S13             GCTCACAACGACACAATGCTGGTCCGGGAACAACAAGAAGTGAAGGCGGGTCAAAAAATA 60
AS13            GCTCACAACGACACAATGCTGGTCCGGGAACAACAAGAAGTGAAGGCGGGTCAAAAAATA 60
AS12            GCTCACAACGACACAATGCTGGTCCGGGAACAACAAGAAGTGAAGGCGGGTCAAAAAATA 60
AS9             GCTCACAACGACACAATGCTGGTCCGGGAACAACAAGAAGTGAAGGCGGGTCAAAAAATA 60
V4              GCTCACAACGACACAATGCTGGTCCGGGAACAACAAGAAGTGAAGGCGGGTCAAAAAATA 60
FGI94           GCTCATAACGACACAATGCTGGTCCGGGAACAACAAGAAGTGAAGGCGGGTCAAAAAATA 60
RB-25           GCCCATAACGACACAATGCTGGTCCGGGAACAACAAGAGGTGAAGGCGGGGCAAAAAATA 60
                ** ** ******************************** *********** *********

SM39            GCCACCATGGGTAGCACCGGTACCAGTTCAGTACGTTTGCATTTTGAAATTCGTTACAAG 120
Db11            GCCACCATGGGTAGCACCGGTACCAGTTCAGTACGTTTGCATTTTGAAATTCGTTACAAG 120
WW4             GCCACCATGGGTAGCACCGGTACCAGTTCAGTACGTTTGCATTTTGAAATTCGTTACAAG 120
ATCC_27592      GCCACCATGGGTAGCACCGGAACCAGTTCAGTACGTTTGCATTTTGAAATTCGTTACAAG 120
568             GCCACCATGGGTAGCACCGGAACCAGTTCAGTACGTTTGCATTTTGAAATTCGTTACAAG 120
A153            GCCACCATGGGTAGCACCGGAACCAGTTCAGTACGTTTGCATTTTGAAATTCGTTACAAG 120
4Rx5            GCCACCATGGGTAGCACCGGAACCAGTTCAGTACGTTTGCATTTTGAAATTCGTTACAAG 120
4Rx13           GCCACCATGGGTAGCACCGGAACCAGTTCAGTACGTTTGCATTTTGAAATTCGTTACAAG 120
S13             GCCACCATGGGTAGCACCGGAACCAGTTCAGTACGTTTGCATTTTGAAATTCGTTACAAG 120
AS13            GCCACCATGGGTAGCACCGGAACCAGTTCAGTACGTTTGCATTTTGAAATTCGTTACAAG 120
AS12            GCCACCATGGGTAGCACCGGAACCAGTTCAGTACGTTTGCATTTTGAAATTCGTTACAAG 120
AS9             GCCACCATGGGTAGCACCGGAACCAGTTCAGTACGTTTGCATTTTGAAATTCGTTACAAG 120
V4              GCCACCATGGGTAGCACCGGAACCAGTTCAGTACGTTTGCATTTTGAAATTCGTTACAAG 120
FGI94           GCCACCATGGGTAGCACCGGAACCAGTTCAGTACGTTTGCATTTTGAAATTCGTTACAAG 120
RB-25           GCAACGATGGGAAGCACCGGAACCAGTTCAGTACGTTTGCATTTTGAAATTCGTTACAAG 120
                ** ** ***** ******** ***************************************

SM39            GGGAAATCCGTAAACCCGCTGCGTTATCTTCCGCAGCGATAGATTGGGCAGAATACGCTG 180
Db11            GGGAAATCCGTAAACCCGCTGCGTTATCTTCCGCAGCGATAGATTGGGCAGAATACGCTG 180
WW4             GGGAAATCCGTAAACCCGCTGCGTTATCTTCCGCAGCGATAGATTGGGCAGAATACGCTG 180
ATCC_27592      GGGAAATCCGTAAACCCGCTGCGTTATCTTCCGCAGCGATAGATTGGGCAGAATACGCTG 180
568             GGGAAATCCGTAAACCCGCTGCGTTATCTTCCGCAGCGATAGATTGGGCAGAATACGCTG 180
A153            GGGAAATCCGTAAACCCGCTGCGTTTTCTTCCGCAGCGATAGATTGGGCAGAATACGCTG 180
4Rx5            GGGAAATCCGTAAACCCGCTGCGTTTTCTTCCGCAGCGATAGATTGGGCAGAATACGCTG 180
4Rx13           GGGAAATCCGTAAACCCGCTGCGTTTTCTTCCGCAGCGATAGATTGGGCAGAATACGCTG 180
S13             GGGAAATCCGTAAACCCGCTGCGTTTTCTTCCGCAGCGATAGATTGGGCAGAATACGCTG 180
AS13            GGGAAATCCGTAAACCCGCTGCGTTTTCTTCCGCAGCGATAGATTGGGCAGAATACGCTG 180
AS12            GGGAAATCCGTAAACCCGCTGCGTTTTCTTCCGCAGCGATAGATTGGGCAGAATACGCTG 180
AS9             GGGAAATCCGTAAACCCGCTGCGTTTTCTTCCGCAGCGATAGATTGGGCAGAATACGCTG 180
V4              GGGAAATCCGTAAACCCGCTGCGTTTTCTTCCGCAGCGATAGATTGGGCAGAATACGCTG 180
FGI94           GGGAAATCCGTAAACCCGCTGCGTTATCTTCCGCAGCGATAGATTGGGCAGAATATGCTG 180
RB-25           GGGAAATCCGTAAACCCGCTGCGTTATCTTCCGCAGCGATAGATTGGACAGAGTGCGTTG 180
                ************************* ********************* **** *   * **

SM39            AGATTCTGCTCGCGGTATCACGGGTAGGAGCAGCATATG 219
Db11            AGATTCTGCTCGCGGTATCACGGGTAGGAGCAGCATATG 219
WW4             AGATTCTGCTCGCGGTATCACGGGTAGGAGCAGCATATG 219
ATCC_27592      TTATTCTGCTCGCGGTATCACGGGTAGGAGCAGCATATG 219
568             T-ATTCTGCTCGCGGTATCACGGGTAGGAGCAGCATATG 218
A153            ATATTCTGCTCGCGGTATCACGGGTAGGAGCAGCATATG 219
4Rx5            ATATTCTGCTCGCGGTATCACGGGTAGGAGCAGCATATG 219
4Rx13           ATATTCTGCTCGCGGTATCACGGGTAGGAGCAGCATATG 219
S13             ATATTCTGCTCGCGGTATCACGGGTAGGAGCAGCATATG 219
AS13            ATATTCTGCTCGCGGTATCACGGGTAGGAGCAGCATATG 219
AS12            ATATTCTGCTCGCGGTATCACGGGTAGGAGCAGCATATG 219
AS9             ATATTCTGCTCGCGGTATCACGGGTAGGAGCAGCATATG 219
V4              ATATTCTGCTCGCGGTATCACGGGTAGGAGCAGCATATG 219
FGI94           ATATTCTGCTCGCGGTATCACGGGTAGGAGCAGCTTATG 219
RB-25           -TATTCTGTTCGCGGTATCACGGGTAGGAGCCGCTTATG 218
                  ****** ********************** ** ****
```

Supplementary Figure S14. Multiple alignment of the untranslated regions of the *rpoS* leader from different *Serratia* strains. The $(AAN)_4$ motif and the A_6 element, essential for the Hfq binding (Soper *et al*., 2010), are highlighted in blue and yellow, respectively. Start codon (grey) and Shine-Dalgarno (underlined) sequences are also shown. Multiple sequence alignments were carried out with ClustalW2 (European Bioinformatics Institute). SM39, *Serratia marcescens* SM39; Db11, *Serratia marcescens* Db11; WW4, *Serratia marcescens* WW4; A153, *Serratia plymuthica* A153; 4Rx5, *Serratia plymuthica* 4Rx5; 4Rx13, *Serratia plymuthica* 4Rx13; S13, *Serratia* sp. S13; *AS13, Serratia plymuthica* AS13; AS12, *Serratia plymuthica* AS12; AS9, *Serratia plymuthica* AS9; ATCC_27592, *Serratia liquefaciens* ATCC_27592; 598, *Serratia proteomaculans* 598; FGI94, *Serratia marcescens* FGI94; V4, *Serratia plymuthica* V4; RB-25, *Serratia fonticola* RB-25.

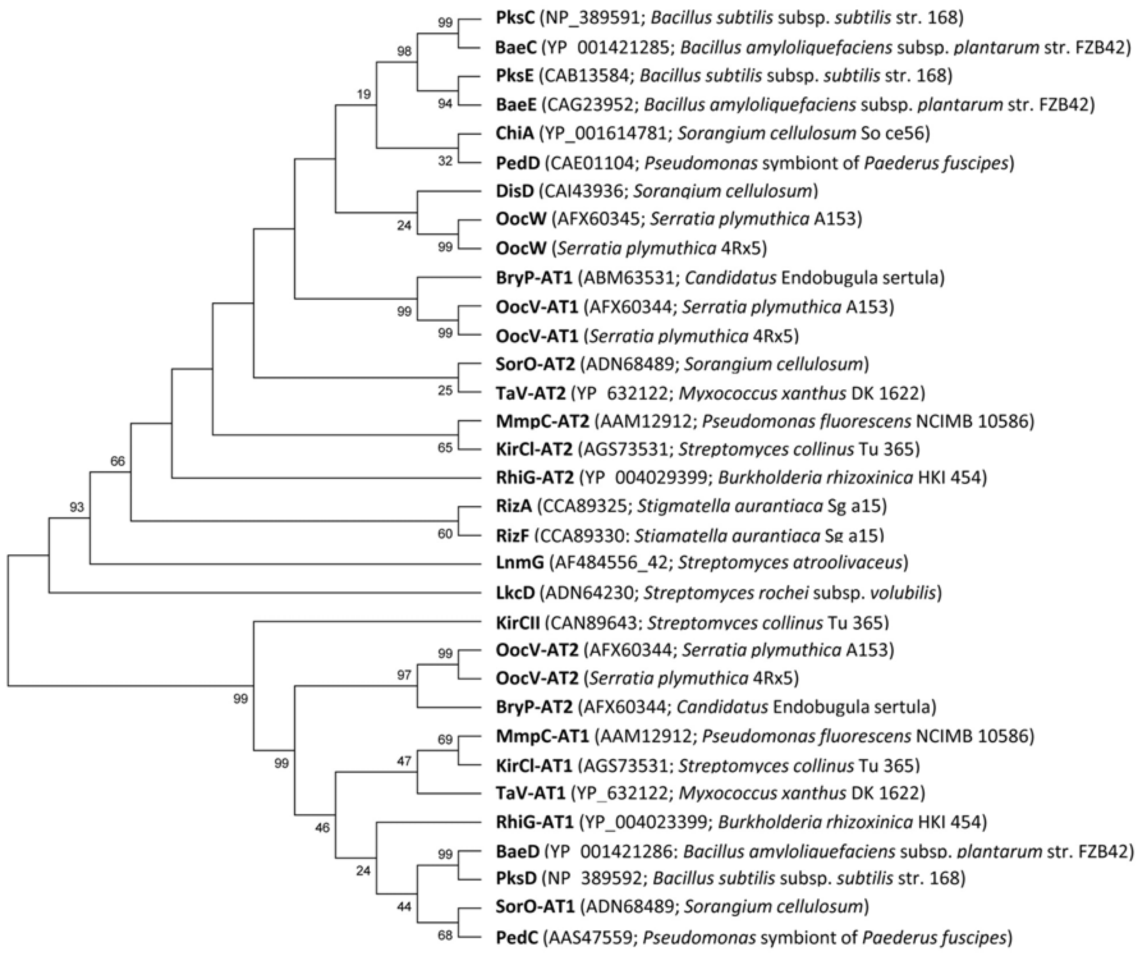

Supplementary Figure S15. Phylogenetic tree of the *trans*-acyltransferase (AT) domains from several *trans*-AT PKS gene clusters. Multiple sequence alignments were carried with ClustalW (European Bioinformatics Institute). The phylogenetic trees were generated with the MEGA software v6 (Tamura *et al*., 2013) using the neighbor-joining method with *P* distance values*.* Bootstrap values (expressed as percentages of 500 replicates) are shown at branch points.

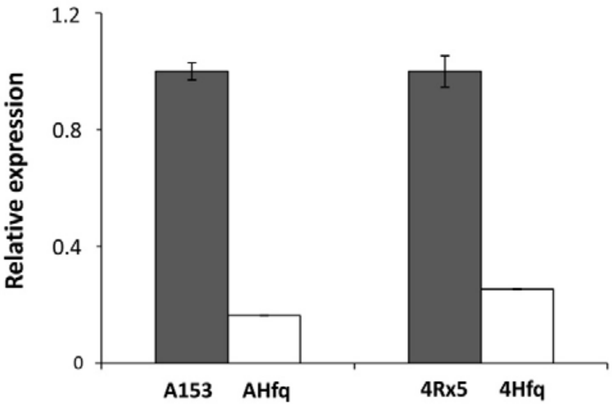

Supplementary Figure S16. Impact of Hfq on *rpoS* transcript levels measured by qPCR. Measurement of *rpoS* transcript levels in *Serratia plymuthica* strains A153 and 4Rx5. The values showed the average expression at stationary phase of growth relative to the wild type expression. Samples for qPCR were taken after growing A153 and 4Rx5 strains in LB medium at 25 ºC for 6 h and 8 h, respectively (see Fig. S10). Data are the mean and standard deviation of three biological replicates.

SUPPLEMENTARY EXPERIMENTAL PROCEDURES

Construction of strains and plasmids

Chromosomal mutants of *Serratia plymuthica* strains were constructed by homologous recombination using derivative plasmids of the suicide vector pKNG101. These plasmids, which are listed in Table S3, were confirmed by DNA sequencing and they carried mutant alleles or in-frame deletions for the replacement of wild type genes in the chromosome. Primers used in this study are listed in Table S4. In all cases, plasmids for mutagenesis were transferred to *S. plymuthica* strains by triparental conjugation using *E. coli* CC118*λpir* and *E. coli* HH26 (pNJ500) as helper. The plasmids for the construction of the in-frame deletions mutants were generated by amplifying the up- and downstream flanking regions of the gene or domain to be deleted. The resulting PCR products were digested with the enzymes specified in Table S3 and ligated in a three-way ligation into pUC18Not, previously cloned into the marker exchange vector pKNG101. The in-frame deletion mutant strains OOA, OOB, OOE, OOK, OOM, OcO, OOP, OOQ, OOT, OOU, OOV, OOV-AT1, OOV-AT2, OocVW and 4SplI were generated using plasmids pMAMV122, pMAMV133, pMAMV68, pMAMV65, pMAMV66, pMAMV123, pMAMV134, pMAMV124, pMAMV71, pMAMV67, pMAMV89, pMAMV86, pMAMV87, pMAMV180 and pMAMV181, respectively. Mutant AT1W was constructed using pMAMV86, and OocW as recipient strain. Alternatively, the 5´ and 3´ flanking regions of *oocE* and *oocU* were directly ligated in a three-way ligation into pKNG101. A153 LacZ was generated by deleting a 1.4 kb region of the *lacZ* gene using plasmid pMAMV112. A153 mutant strains defective in *oocW, hfq* and *rpoS* were constructed through the insertion of a kanamycin resistance cassette using plasmids pMAMV88, pMAMV160 and pMAMV113, respectively. The *hfq* gene and its genomic context in A153 and 4Rx5 are 100% identical and both strains AHfq and 4Hfq were generated using pMAMV160. For the construction of the strains OocJ-ACP, OocS-TE and OocS-C, point mutations in the ACP_L, TE and NRPS-C domains were generated

by overlapping PCR. The final PCR product was cloned into pUC18Not previously to be cloned into the NotI site of pKNG101 and the plasmids pMAMV99, pMAMV100 and pMAMV101 were used for the construction of the strains OocJ-ACP, OocS-TE and OocS-C, respectively. All relevant mutations and were confirmed by PCR and sequencing.

For the construction of the complementing plasmids, the genes were amplified using primers described in Table S4 and cloned into pTA100 or pTRB30. To generate pAT1, pAT2 and pAT3, the catalytic serine present in the acyltransferase domains OocV-AT1, OocV-AT2 and OocW-AT3 was replaced by alanine by overlapping PCR. All the inserts were confirmed by PCR and sequencing. For the construction of the transcriptional fusions to *´lacZ*, the promoter regions of *oocG* and *oocJ* were amplified using primers described in Table S4 and subsequently cloned into pMP220. Complementing plasmids were transformed into *Serratia* and *Dickeya* strains by electroporation.

REFERENCES

1. Berg, G., Roskot, N., Steidle, A., Eberl, L., Zock, A., and Smalla, K. (2002) Plant-dependent genotypic and phenotypic diversity of antagonistic rhizobacteria isolated from different *Verticillium* host plants. Appl Environ Microbiol 68: 3328-3338.

2. Demarre, G., Guérout, A.M., Matsumoto-Mashimo, C., Rowe-Magnus, D.A., Marlière, P., and Mazel, D. (2005) A new family of mobilizable suicide plasmids based on broad host range R388 plasmid (IncW) and RP4 plasmid (IncPalpha) conjugative machineries and their cognate *Escherichia coli* host strains. Res Microbiol 156: 245-255.

3. Dennis, J. J., and Zylstra, G.J. (1998) Plasposons: modular self-cloning minitransposon derivatives for rapid genetic analysis of Gram-negative bacterial genomes. Appl Environ Microbiol 64: 2710-2715.

4. Grinter, N.J. (1983) A broad host range cloning vector transposable to various replicons. Gene 21: 133-143.

5. Fineran, P.C., Blower, T.R., Foulds, I.J., Humphreys, D.P., Lilley, K.S., and Salmond G.P.C. (2009) The phage abortive infection system, ToxIN, functions as a protein-RNA toxin-antitoxin pair. Proc Natl Acad Sci U.S.A. 106: 894-899.

6. Frazer, K.A., Pachter, L., Poliakov, A., Rubin, E.M. and Dubchak, I. (2004) VISTA: computational tools for comparative genomics. Nucleic Acids Res 32: W273-279.

7. Herrero, M., de Lorenzo, V., and Timmis, K.N. (1990) Transposon vectors containing nonantibiotic resistance selection markers for cloning and stable chromosomal insertion of foreign genes in Gram-negative bacteria. J Bacteriol 172: 6557-6567.

8. Hökeberg, M., Gerhardson, B., and Johnsson, L. (1997) Biological control of cereal seed-borne diseases by seed bacterization with greenhouse-selected bacteria. Eur J Plant Pathol 103: 25-33.

9. Kaniga, K., Delor, I., and Cornelis, G.R. (1991) A wide-host range suicide vector for improving reverse genetics in Gram-negative bacteria: inactivation of the *blaA* gene of *Yersinia enterocolitica.* Gene 109: 137-141.

10. Matilla, M.A., Stöckmann, H., Leeper, F.J., and Salmond, G.P.C. (2012) Bacterial biosynthetic gene clusters encoding the anti-cancer haterumalide class of molecules: biogenesis of the broad spectrum antifungal and anti-oomycete compound, oocydin A. J Biol Chem 287: 39125-39138.

11. Matilla, M. A., and Salmond, G.P.C.(2014) The viunalikevirus, bacteriophage ϕMAM1, is a broad host-range, high efficiency generalised transducing phage that infects environmental and clinical isolates of the enterobacteria*, Serratia* and *Kluyvera*. Appl Environ Microbiol 80: 6446-6457.

12. Maurhofer, M., Reimmann, C., Schmidli-Sacherer, P., Heeb, S., Haas, D., and Défago, G. (1998) Salicylic acid biosynthetic genes expressed in *Pseudomonas*

fluorescens strain P3 improve the induction of systemic resistance in tobacco against tobacco necrosis virus. Phytopathology 88: 678-84.

13. McClean, K.H., Winson, M.K., Fish, L., Taylor, A., Chhabra, S.R., Camara, M., *et al.* (1997) Quorum sensing and *Chromobacterium violaceum*: exploitation of violacein production and inhibition for the detection of *N*-acylhomoserine lactones. Microbiology 143: 3703-3711.

14. Poulter, S., Carlton, T. M., Su, X., Spring, D.R., and Salmond, G.P.C. (2010) Engineering of new prodigiosin-based biosensors of Serratia for facile detection of short-chain *N*-acyl homoserine lactone quorum-sensing molecules. Environ Microbiol Rep 2: 322-328.

15. Pritchard, L., Humphris, S., Baeyen, S., Maes, M., Van Vaerenbergh, J., Elphinstone, J., Saddler, G., and Toth, I. (2013a). Draft genome sequences of four *Dickeya dianthicola* and four *Dickeya solani* strains. Genome Announc 1(4). pii: e00087-12.

16. Pritchard, L., Humphris, S., Saddler, G.S., Elphinstone, J.G., Pirhonen, M., and Toth, I.K. (2013b) Draft genome sequences of 17 isolates of the plant pathogenic bacterium *Dickeya*. Genome Announc 1(6). pii: e00978-13.

17. Reimmann, C., Ginet, N., Michel, L., Keel, C., Michaux, P., Krishnapillai, V. *et al*. (2002) Genetically programmed autoinducer destruction reduces virulence gene expression and swarming motility in *Pseudomonas aeruginosa* PAO1. Microbiology 148: 923-32.

18. Soper, T., Mandin, P., Majdalani, N., Gottesman, S., and Woodson, S.A. (2010) Positive regulation by small RNAs and the role of Hfq. Proc Natl Acad Sci USA 107: 9602-9607.

19. Spaink, H.P., Okker, R.J.H., Wijffelman, C.A., Pees, E., and Lugtenberg, B.J.J. (1987) Promoters in the nodulation region of the *Rhizobium leguminosarum* Sym plasmid pRL1JI. Plant Mol Biol 9: 27-39.

20. Strobel, G., Li, J.Y., Sugawara, F., Koshino, H., Harper, J., and Hess, W.M. (1999) Oocydin A, a chlorinated macrocyclic lactone with potent anti-oomycete activity from *Serratia marcescens*. Microbiology 145: 3557-3564.

21. Tamura, K., Stecher, G., Peterson, D., Filipski, A., and Kumar, S. (2013) MEGA6: Molecular Evolutionary Genetics Analysis Version 6.0. Mol Biol Evol 30: 2725-2729.

22. Woodcock, D.M., Crowther, P.J., Doherty, J., Jefferson, S., DeCruz, E., Noyer-Weidner, M., *et al*. (1989) Quantitative evaluation of *Escherichia coli* host strains for tolerance to cytosine methylation in plasmid and phage recombinants. Nucleic Acids Res 17: 3469-3478.
